# Fast Charge Separation in Distant Donor–Acceptor Dyads Driven by Relaxation of a Hot Excited State

**DOI:** 10.1021/acs.jpcc.2c05754

**Published:** 2022-10-20

**Authors:** Zimu Wei, Abbey M. Philip, Wolter F. Jager, Ferdinand C. Grozema

**Affiliations:** Department of Chemical Engineering, Delft University of Technology, Van der Maasweg 9, 2629 HZDelft, The Netherlands

## Abstract

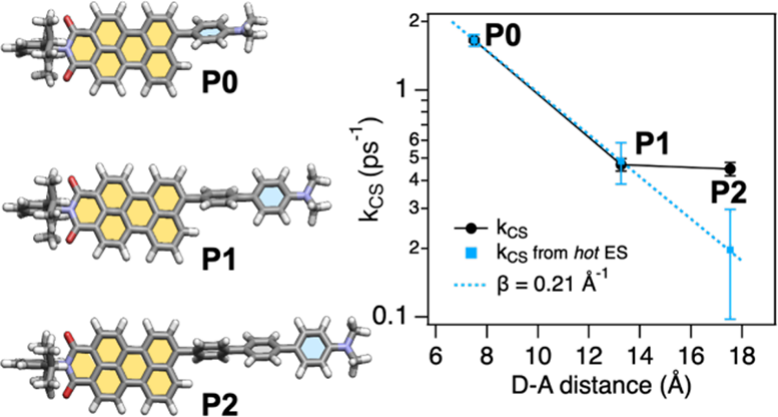

A series of three perylenemonoimide-*p*-oligophenylene-dimethylaniline molecular dyads undergo photoinduced charge separation (CS) with anomalous distance dependence as a function of increasing donor–acceptor (DA) distances. A comprehensive experimental and computational investigation of the photodynamics in the donor–bridge–acceptor (DBA) chromophores reveals a clear demarcation concerning the nature of the CS accessed at shorter (bridgeless) and longer DA distances. At the shortest distance, a strong DA interaction and ground-state charge delocalization populate a hot excited state (ES) with prominent charge transfer (CT) character, via Franck–Condon vertical excitation. The presence of such a CT-polarized hot ES enables a subpicosecond CS in the bridgeless dyad. The incorporation of the *p*-oligophenylene bridge effectively decouples the donor and the acceptor units in the ground state and consequentially suppresses the CT polarization in the hot ES. Theoretically, this should render a slower CS at longer distances. However, the transient absorption measurement reveals a fast CS process at the longer distance, contrary to the anticipated exponential distance dependence of the CS rates. A closer look into the excited-state dynamics suggests that the hot ES undergoes ultrafast geometry relaxation (τ < 1 ps) to create a relaxed ES. As compared to a decoupled, twisted geometry in the hot ES, the geometry of the relaxed ES exhibits a more planar conformation of the *p*-oligophenylene bridges. Planarization of the bridge endorses an increased charge delocalization and a prominent CT character in the relaxed ES and forms the origin for the evident fast CS at the longest distance. Thus, the relaxation of the hot ES and the concomitantly enhanced charge delocalization adds a new caveat to the classic nature of distance-dependent CS in artificial DBA chromophores and recommends a cautious treatment of the attenuation factor (β) while discussing anomalous CS trends.

## Introduction

Inspired by nature, there is a strong interest in developing artificial systems for driving photochemical reactions, producing electrical output, or deriving stimulus-responsive photoswitches.^1−6^ In these natural^7^ and artificial systems,^2,4,8^ charge transfer (CT) processes play a quintessential role and thus draw a major interest from a scientific and technological perspective.^9^ To tailor the CT properties for a specific application,^10,11^ it is crucial to understand the fundamental nature of the excited state (ES), the precursor state for a CT process. Many such examples of synthetic control over the nature of the ES have been achieved by artificial donor–bridge–acceptor (DBA) chromophores that combine electron-donating and electron-accepting groups with complementary (opto)electronic character.^3,10,11^

In a typical DBA system, the photoinduced charge separation (CS) is dictated by the thermodynamic^12^ and kinetic^13^ parameters including the free energy (Δ*G*_CS_^0^), reorganization energy (λ) and electronic coupling (*V*). The thermodynamic feasibility (Δ*G*_CS_^0^) for efficient CS relies on the D/A redox potentials, molecule distances (*R*_DA_), and solvent polarity.^12^ The latter two also influence the reorganization energy (λ). The electronic coupling (*V*) strongly depends on the nature of the molecular bridge, the D/A components, their relative spatial orientation,^14−17^ and distances (*R*_DA_).^13,18−21^ Because of the exponential radial character of electronic wave functions, the electronic coupling (*V*) is expected to decay exponentially with increasing donor–acceptor distances (*R*_DA_).

Consequently, the CS kinetic rates (*k*_CS_) in DBA systems generally follows an exponential distance dependence in the tunneling regime at short distances (<20 Å),^22^*k*_CS_ = *k*_0_e^–β*R*_DA_^ wherein *k*_0_ is a kinetic prefactor and β is the attenuation factor that characterizes the capability of the intervening bridge to transfer charge. The attenuation factor β has been perceived to be a bridge-dependent parameter, and the reported values of the attenuation factor span a wide range between fully conducting (β < 0.1 *Å*^–1^)^23−25^ and highly insulating (β > 0.5 *Å*^–1^) bridges.^26,27^ It has been observed that for the same π-conjugated bridge, β values can be considerably dissimilar in different DBA systems, and many mechanisms have been proposed to account for the anomalous distance dependence observed experimentally.^28,29^ For instance, it is widely recognized that switching from coherent tunneling (superexchange) to incoherent hopping is responsible for the shallow distance dependence and small β values at a long donor–acceptor (DA) distance (up to 40 Å).^19,30^ In the frame of the superexchange mechanism, nonexponential distance dependence has been observed and attributed to a crossover from the inverted to normal CS regime, owing to high reorganization energy with an increasing distance.^31,32^ At short DA distances (<20 Å), however, a sharp decrease in the CS rate with the distance is generally expected.^33^ Only a few exceptions have been reported mostly due to system-specific mechanisms, such as the initial state delocalization,^34^ strong solvent polarity,^35^ and resonant bridge states.^36^ Therefore, a comprehensive understanding on mechanisms for an anomalous CS trend at a short distance will pave the way to customize DBA chromophores for specific applications with a broad choice of molecular lengths.

**Figure 1:**
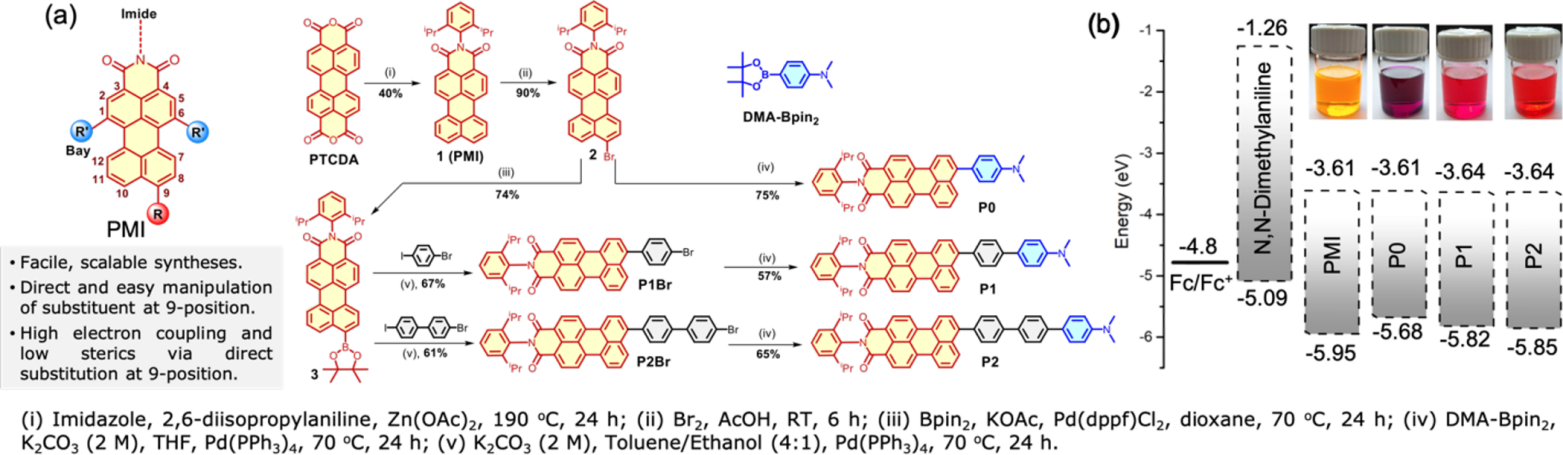
(a) Modular structures of **PMI**-based chromophores and synthesis scheme and (b) energy levels of the reference (**PMI**) and DBA derivatives (**P0**, **P1**, and **P2**) against vacuum.

Herein, we explore the nature of the distance-dependent CS in a series of short DBA dyads (**P0**, **P1**, and **P2**) wherein a perylenemonoimide (**PMI**) acceptor is connected to a dimethylaniline (**DMA**) donor via intervening *p*-oligophenylene bridges (*n* = 0, 1, and 2, Figure 1). The excited-state photodynamics within the DBA compounds was evaluated using steady-state optical, ultrafast transient absorption, and theoretical investigations. Evidence from the comprehensive experimental and computational studies indicates an anomalous deviation from the exponential attenuation of the CS rates at longer distances (for **P2**) as compared to the shorter distances (**P0** and **P1**). The origin of the deviation was rationalized by comparing the charge delocalization in the excited states (hot and relaxed ES)^37^ as compared to the ground-state geometries. The comparison suggests that the geometry relaxation of the hot ES results in a more planar and electronically coupled relaxed ES in **P2** that exhibits an enhanced charge delocalization and CT character. This, in turn, increases the rate of charge separation from the relaxed ES and creates a deviation from the anticipated nature of distance dependence of CS rates at longer distances. Thus, charge delocalization adds a new caveat to the nature of distance-dependent charge separation and stresses the importance of treating the attenuation factor β as a system-specific parameter while interpreting anomalous CS trends in distant DBA architectures.

## Methods

### Experimental Methods

The synthesis scheme and the structures of the reference and the DBA derivatives are depicted in Figure 1a, and the complete characterization is provided in the Supporting Information. The electrochemical characterization of **PMI** and the DBA derivatives was carried out using cyclic voltammetry (V vs Fc/Fc^+^) in a chloroform:acetonitrile (2:3) mixture (Figures S1 and S2). Absorption and photoluminescence measurements were performed in a PerkinElmer Lambda 40 UV–vis spectrophotometer and a Horiba Jobin Yvon SPEX Fluorolog 3 spectrophotometer, respectively. Fluorescence lifetimes were recorded on an Edinburgh LifeSpec-ps spectrometer with a fixed excitation wavelength of 404 nm. Pump-probe fs-TA measurements were performed by first exciting the sample with a pump beam generated by a YB-KGW oscillator. The pump wavelength was tuned by sending the fundamental beam (1028 nm) through an optical parametric amplifier (Light Conversion, ORPHEUS-PO15F5HNP1). Subsequently, a broadband white light (450–915 nm) was used to probe the transient absorption of the excited sample at a series of delay times. With a commercial TA spectrometer (HELIOS, Ultrafast Systems), the change in the absorption spectrum, Δ*A*, was recorded as a function of time. The resulting TA spectrum was analyzed by global and target analysis using Glotaran.^38^ More details regarding the experimental methods are provided in the Supporting Information.

### Theoretical Calculations

Molecular and electronic structure calculations were performed using the Amsterdam Density Functional (ADF) software package.^39^ All calculations were computed at the density functional theory (DFT) level of theory using a range-separated CAM-B3LYP functional with the double-zeta plus polarization (DZP) basis set in vacuum if not specified otherwise. The CAM-B3LYP functional was chosen because it offers a good description for the energetics of excited states when handling charge transfer states and related Coulomb interactions.^40,41^ With time-dependent density functional theory (TDDFT), the optimized ground-state geometries were used to calculate the vertical absorption spectra. Based on such spectra, the hot excited states corresponding to the vertical excitation of the PMI acceptor were derived. To further study the relaxed excited states, the excited-state geometries were optimized using the ground-state gradients and the gradients of the TDDFT excitation energy for the lowest allowed transition.^42^ To quantify the charge delocalization in the hot and relaxed excited states, the difference in charge distribution between the ground state and the excited state for a given geometry was calculated. In detail, the charge distribution in the ground state was derived from the Mulliken^43^ charges on the donor, the acceptor, and each intervening phenyl unit. The charge distribution in the excited state was summed over the different contributions to the excited state from all single orbital transitions with a contribution larger than 2%.

## Results and Discussion

Perylenemonoimide (**PMI**) belongs to the family of rylene chromophores, which are widely known for their promising optical features, excellent photo(chemical) stability, and electron-deficient nature.^44−46^**PMI** was chosen as the electron acceptor owing to its facile, scalable syntheses and the ease of direct functionalization at the 9-position as reported earlier (Figure 1a).^47−54^*N*,*N*-Dimethylaniline (**DMA**) was utilized as the electron donor owing to its strong electron-donating nature as well as its proton sensitivity. To construct the DBA scaffolds, **PMI** and **DMA** were directly connected at the 9-position with an intervening *p*-oligophenylene bridge of varying lengths. A direct connection with a rigid bridge not only defines the distance but also affords sufficient electronic coupling between the distant D/A units. Additionally, the substitution at the 9-position of **PMI** renders a minimal steric block between D and A units, as opposed to the large steric congestion at the bay region. Herein, unsubstituted perylenemonoimide (**PMI**) acts as a model reference for **P0** (no bridge), **P1** (phenyl bridge), and **P2** (biphenyl bridge) DBA derivatives. Though **PMI** and **DMA** units are connected via a rigid, nonfunctionalized *p*-oligophenylene bridge, the DBA compounds possess adequate solubility in weakly polar toluene, moderately polar tetrahydrofuran (THF), and polar benzonitrile (Bzn) solvents used for the spectroscopic studies. The derivatives **P0** exhibited sufficient solubility in nonpolar hexane as compared to **P1** and **P2**, which were insoluble in that solvent.

The standard values of the redox potentials are reported in Table S1 along with the HOMO/LUMO energy levels versus vacuum (Figure 1b). The reference **PMI** exhibits a first reduction at *ca*. −1.38 V and oxidation at *ca.* 0.98 V.^55^ The electrochemical reduction of the perylene core in the DBA derivatives becomes gradually more facile as the distance between **DMA** and the perylene core increases in **P0** (*ca.* −1.39 V), **P1** (*ca*. −1.38 V), and **P2** (*ca.* −1.36 V), and a reverse trend is observed for the oxidation of the perylene core. The observed changes in the redox properties translate into HOMO–LUMO energy gap (*E*_g_) tuning. The *E*_g_ of **P0** is 0.27 eV smaller than the unsubstituted **PMI**, implying a strong ground-state interaction among the **PMI** and **DMA** donor.^56^ The HOMO–LUMO energy gap increases as the **PMI** and **DMA** groups are further separated in **P1**/**P2**, and this is clearly reflected in the systematic tuning of the optical color of the solutions (Figure 1b).

### Steady-State Optical Properties

**Figure 2:**
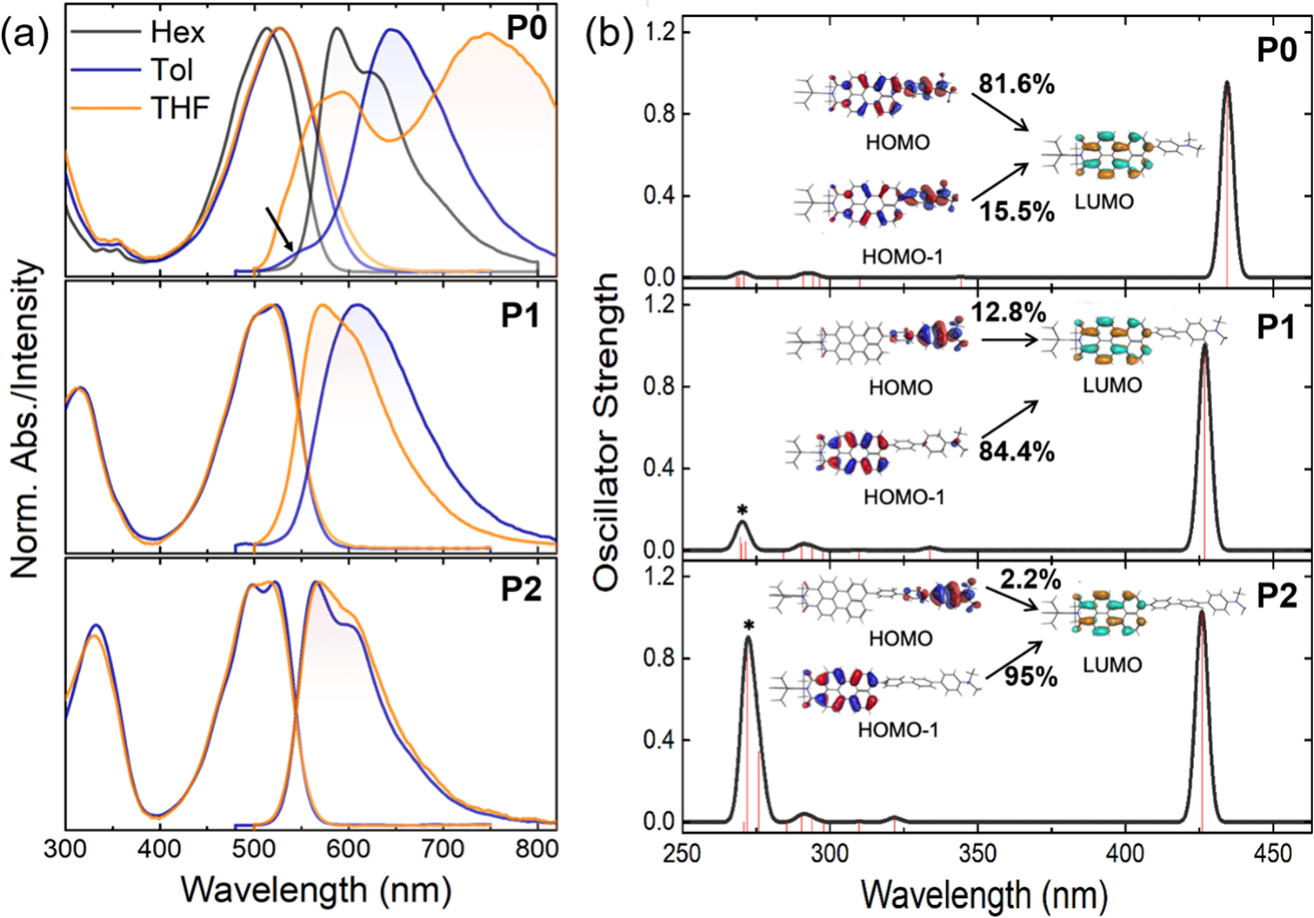
(a) Normalized UV–vis absorbance (solid lines) and fluorescence emission (dashed lines, ex = 490 nm) spectra in solvents of varying polarities and (b) simulated optical excitation for **P0** (top), **P1** (middle), and **P2** (bottom) calculated using TD-DFT with CAM-B3LYP/DZP. The arrow in the emission spectra of **P0** highlights the diffuse emission band in toluene. *Bands appearing at a high energy indicate the mixed transition involving the *p*-oligophenylene bridge.

To probe the photoexcited-state properties and their distance dependence, steady-state UV–vis absorption and fluorescence (Figure 2) of the DBA compounds were measured in solvents of varying polarities (Table 1 and Figures S3–S9). The UV–vis absorption of the **PMI** in toluene is characterized by typical singlet π–π* transitions of the perylene chromophore in the 400–550 nm range and depicts negligible change with the increasing solvent polarity (Figure S4).^57^ The DBA derivative **P0** exhibits a broad, structureless UV–vis absorption in toluene and a concomitant bathochromic shift upon increasing the solvent polarity (Figure 2a). The broad, featureless UV–vis absorption in **P0** as compared to the reference **PMI** and the strong solvent polarity dependence suggest that the Franck–Condon (FC) vertical excitation is accompanied by a partial charge transfer (CT) from the D to A unit. Thus, the hot excited state (ES)^37^ in **P0**, formed upon FC vertical excitation, is delocalized showing CT character.^58^ Upon introducing the *p*-oligophenylene bridge between the **PMI** and **DMA** groups, a vibronically structured UV–vis absorption is observed in **P1** and **P2**, analogous to **PMI** in toluene. Although the UV–vis absorption of the **P1** and **P2** still deviates from that of **PMI** (Figure S3), the deviation becomes much smaller than that of **P0**, implying that the degree of delocalization in hot ES is gradually suppressed with the increasing bridge length. This is evidently observed in the form that the magnitude of the solvatochromic bathochromic shift in **P1/P2** is minuscule as compared to **P0**. This further suggests that the CT polarization in the hot ES of **P1/P2** is small and diminished for longer bridge lengths.

**Table 1:** Optical Properties of the Reference and DBA Compounds in Solvents of Varying Polarities^a^

	solvent	λ_abs_ (nm)	λ_em_ (nm)	Stokes shift^*a*^ [Δλ] (nm)	ϕ_F_^*b*^	ϕ_F_^*c*^	FE	*τ*_F_ (ns)^*f*^
**PMI**	toluene	507	530	23	0.88	---^*d*^	---^*d*^	4.54
	THF	502	536	34	0.91	---^*d*^	---^*d*^	4.87
	Bzn	511	550	39	0.83	---^*d*^	---^*d*^	4.93
**P0**	hexane	513	587	74	0.81	---^*d*^	---^*d*^	3.92
	toluene	527	553^*e*^, 644	117	0.60	---^*d*^	---^*d*^	3.44^*g*^
	THF	527	593, 748	221	0.12	0.86	7.2	3.35,^*h*^ 0.76^*i*^
	Bzn	545	611, 778^*e*^	233	0.05	0.84	16.8	3.89^*j*^
**P1**	toluene	523	608	85	0.70	---^*d*^	---^*d*^	3.55
	THF	518	572	54	0.027	0.86	32	3.77
	Bzn	529	590	61	0.018	0.81	45	4.18
**P2**	toluene	522	566	44	0.78	---^*d*^	---^*d*^	3.55
	THF	516	569	53	0.055	0.839	16.2	3.67
	Bzn	526	591	65	0.015	0.76	51	4.04

a Note: ^*a*^Stokes shifts with respect to the redshifted band. Fluorescence quantum yield in ^*b*^neutral and ^*c*^protonated states (using 48% HBr solution), ^*d*^no protonation attempted, and the ^*e*^diffuse band. FE = fluorescence enhancement in the protonated *versus* nonprotonated state; ^*f*^excitation at 404 nm. The emission lifetimes were monitored around the emission maxima unless otherwise mentioned; emission monitored at ^*g*^640, ^*h*^600, ^*i*^750, and ^*j*^610 nm. Photophysical measurements of **PMI**, **P1**, and **P2** in hexane were not possible owing to the low solubility.

To gain a more detailed picture of the hot ES of the DBA compounds, DFT calculations were performed using the Amsterdam Density Functional (ADF) theory package using the range-separated CAM-B3LYP functional with the double-zeta plus polarization (DZP) basis set (Figure 2b and Table S4).^39^ The simulated absorption spectra of the reference **PMI** reveal a dominant HOMO⃗LUMO (∼416 nm) transition localized on the perylene core (π–π*) as the main band with a considerable oscillator strength (Figure S10). For DBA compounds, the nature of the excited states exhibits a marked distance dependence as compared to **PMI**. In **P0**, the principal band at 434 nm has prominent contributions from HOMO⃗LUMO (81.6%) and HOMO-1→LUMO (15.5%) CT-type transitions. In **P1**, the fundamental absorption at 427 nm mainly consists of π–π*-type HOMO-1→LUMO (84.4%) and a much smaller CT-type HOMO⃗LUMO (12.8%) transition. This trend is further apparent in **P2**, wherein a further reduction in the CT character (HOMO-1→LUMO; 2.2%) is observed for the fundamental absorption band at 426 nm. Additionally, with the increasing length of the bridge in **P1** and **P2**, a new band (with considerable oscillator strengths) emerges in the near-UV region, similar to that observed in UV–vis spectra. These high-energy absorption bands in **P1** and **P2** arise from complex mixed transitions involving the *p*-oligophenylene bridge (see Figure S11).

The distance-dependent CT character of the hot ES in DBA derivatives is established from the UV–vis experiments and molecular simulations. The nature of the relaxed ES and its distance dependence can further be established from the fluorescence measurements in solvents of varying polarities (Figure 2a and Figures S3–S9). The fluorescence spectrum of the reference **PMI** in toluene, THF, and Bzn centers around 500–750 nm with a mirror image symmetry to the UV–vis absorption. Marginal solvent dependence of radiative emission for **PMI** suggests a low impact of the solvent environment on the bright local excited state and a small dipole moment change upon FC vertical excitation (Figure S4b and Table S3).^59^ Contrarily, **P0** depicts an interesting and complex emission profile with respect to the solvent continuum. **P0** in nonpolar hexane depicts a characteristic **PMI**-like emission with a high quantum efficiency (ϕ_F_ ≈ 0.81). Changing the solvent to weakly polar toluene renders a dual emission character at 550 (weak band) and 644 nm (intense and broad) with a considerable Stokes shift (Δλ = 117 nm) and a moderate decrease in quantum yields (ϕ_F_ ≈ 0.60). Increasing the polarity to THF leads to a clear dual emission with an increased Stokes shift (Δλ = 221) for the long-wavelength band as well as a low fluorescence quantum yield (ϕ_F_ ≈ 0.12).^48,60^ Increasing the solvent polarity to Bzn further quenches the fluorescence emission for **P0** (ϕ_F_ ≈ 0.05). Herein, the redshifted band becomes diffuse, and the residual emission becomes prominent from the relaxed ES with minor CT polarization. To assess the dipole moment difference between the ground and excited state, the solvatochromic method was utilized (see the SI for details).^61,62^ The assessment of the ground- (μ_g_ = 2.92 D) and excited-state dipole (μ_e_ = 21.24 and 30.40 D) moments for **P0** reveal (i) a large dipole moment difference (Δμ = 18.32 and 27.48 D) between the ground and excited state and (ii) at least two different dipole moments for the excited state indicating the presence of excited states with a distinct nature of charge distribution.^63,64^

The longer DBA derivatives, **P1** (ϕ_F_ ≈ 0.70, Δλ = 85 nm) and **P2** (ϕ_F_ ≈ 0.78, Δλ = 44 nm), in toluene exhibit an intense fluorescence emission and a narrow Stokes shift as compared to **P0**. With the increasing solvent polarity, **P1**/**P2** (THF and Bzn) exhibits a marginal solvatochromic shift but a significantly quenched fluorescence emission (Figure 2a). The marginal solvatochromic change suggests a smaller dipole moment change upon FC vertical excitation for **P1** (Δμ = 16.34) and **P2** (Δμ = 15.17 D) as compared to **P0** (Table S3). Notably, all three compounds exhibit much larger changes in the dipole moment than the reference **PMI** (Δμ = 7.24 D), indicating a larger degree of delocalization in the relaxed ES of these DBA derivatives.

Accounting for the solvent polarity, **P0**–**P2** exhibit a very systematic fluorescence trend with marginal quenching in weakly polar toluene (see Figure S3b and Table 1). Since the free energy for charge separation (Δ*G*_CS_^0^ > 0) is not favorable for the DBA derivatives in toluene (see Table S5), the systematic fluorescence quenching can be explained by the partial CT polarization in the relaxed ES of **P0**–**P2** that changes as a function of the distance. Contrarily, the fluorescence emission of **P0**–**P2** in (moderately) polar THF/Bzn exhibits drastic quenching (Figure 2a and Figure S3d) and can arise from an interplay of partial and full CS processes, which becomes favorable (Δ*G*_CS_^0^ < 0) in polar solvents (see Table S5).

The ground-state CT mixing and its extent can be further confirmed by the UV–vis absorption and fluorescence emission under protonated conditions. Protonation of the dimethylamino group counters the electron-donating nature of the dimethylamino group and thereby ceases the CT and CS processes. The protonation of **P0**–**P2** in THF/Bzn gives rise to a more **PMI**-like absorption with a distinct hypsochromic shift as compared to the neutral state (Figure S12). The magnitude of the hypsochromic shift in the UV–vis absorption of the protonated compounds scales as the distance between **PMI** and **DMA**, with **P0** exhibiting the most prominent change, **P1** exhibiting moderate change, and **P2** the least change as a function of the DA distance. This observation confirms the distance-dependent CT polarization in **P0**–**P2**, in line with the UV–vis absorption and TD-DFT simulations (Figure S12). Concurrently, the fluorescence emission for protonated **P0**–**P2** is activated from the bright excited state with quantum efficiencies similar to **PMI** (Figure S12). The recovery of the fluorescence emission upon protonation for **P0**–**P2** suggests that the quenched fluorescence emission in highly polar solvents stems from the formation of radical ion-pair intermediates by photoinduced CS.

### Femtosecond Transient Absorption

**Figure 3:**
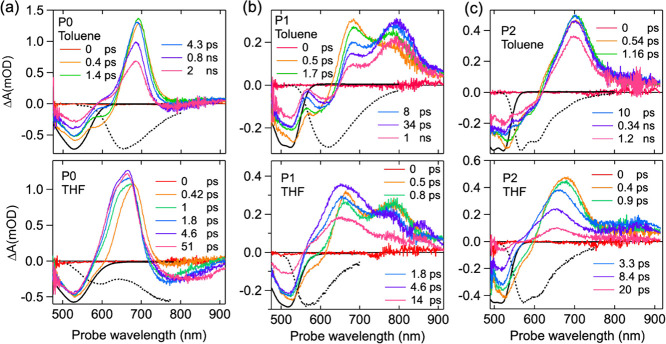
Transient absorption spectra of (a) **P0** (ex, 530 nm), (b) **P1** (ex, 500 nm), and (c) **P2** (ex, 500 nm) in toluene (top) and THF (bottom). The inverted steady-state absorption spectra and emission spectra are included as a black solid line and a black dotted line, respectively.

To gain insights into the dynamics of the excited state in **P0**–**P2**, their femtosecond transient absorption (TA) spectra were measured upon predominantly exciting the **PMI** acceptor at 530 nm for **P0** or at 500 nm for **P1** and **P2**. To distinguish the ES from the CS state in THF, the spectral features of the ES were determined by the TA spectra in nonpolar hexane or acidic solution, where the formation of the CS state is expected to be nonexistent. The TA spectra of **P0** in hexane, toluene, and THF along with the inverted steady-state absorption and emission spectra are depicted in Figure 3a and Figure S14a. After photoexcitation at 530 nm, the TA spectrum in hexane (Figures S14a) is dominated by the local excited (LE) state PMI* as expected and exhibits the long-lived features of the ground-state bleach (GSB, at ∼515 nm), stimulated emission (SE, at ∼585 nm), and a strong induced absorption, peaked at 690 nm due to the S_1_ → S_*n*_ excited-state absorption (ESA) of PMI*.^65^ Upon increasing the solvent polarity to weakly polar toluene, additional spectral features emerge as a positive shoulder between 590 and 640 nm and a minor negative signal at ∼800 nm (Figure 3a). Both features become much more pronounced in THF on an ultrafast timescale (<1 ps). It has been reported that the absorption maximum of the perylenemonoimide radical anion (**PMI**^•–^) ranges from 588^66^ to 640 nm.^67,68^ Thus, the induced absorption between 590 and 640 nm is ascribed to the formation of **PMI**^•–^. It is plausible that the fast relaxation of the ES of PMI* in toluene leads to polarization and creation of a partial CT character, whereas a full CS radical ion-pair state is not observed owing to the unfavorable free energy for charge separation (Δ*G*_CS_^0^ > 0, Table S5). Contrarily, a concurrent formation of a well-defined CS state with redshifted stimulated emission and a radical anion (**PMI**^•–^) is apparent in THF as the free energy is favorable for charge separation (Δ*G*_CS_^0^ < 0). The emissive nature of the CS state apparently suggests that the degree of charge separation in **P0** is fractional and does not reach unity in moderately polar THF.^64^ A larger degree of CS is expected in more polar solvents, and partial evidence comes from the measurements of steady-state fluorescence emission in highly polar Bzn/DMSO where a near-quantitative quenching of the redshifted emission is observed (Figures S5 and S8).^69^

To understand the excited-state dynamics in **P1** and **P2**, the TA spectra were measured in toluene, neutral, and protonated THF solutions (Figure 3b,c and Figure S14b,c). As shown in Figure 3b, the TA spectrum of **P1** in toluene at 0.5 ps displays the GSB (∼530 nm), SE (∼580 nm), and ESA (∼680 nm) of perylene excited-state PMI* as well as an additional absorption peak at ∼790 nm. Subsequently, the decay of PMI* leads to the further growth of an additional absorption peak with a redshift (∼30 nm) in the SE. Herein, only a marginal CT polarization and no CS processes are anticipated for **P1** in toluene as established by the steady-state optical measurements and the unfavorable free energy for CS, respectively. Therefore, the absorption peak at ∼790 nm can be attributed to the CT polarization since the delocalization of the ES of PMI* may extend onto the phenyl bridge and the **DMA** donor leading to additional absorption features. Accordingly, this relaxation of the initial excited state gives rise to the redshift in the SE.

With the increased solvent polarity, the TA spectrum of **P1** in THF shows that the absorption peaks of both the initial and delocalized excited states of **PMI** transform into the absorption of the radical anion **PMI**^•–^ within a few picoseconds (Figure 3b). As a result, the SE of PMI* at ∼600 nm is no longer observable at 1.8 ps. Since the **DMA** radical cation (**DMA**^•**+**^) absorbs at 475 nm,^58^ the small peak at ∼850 nm is likely to be associated with the long-wavelength absorption of **PMI**^•–^ as reported earlier by spectroelectrochemistry.^66^ Following the fast CS process, the charge recombination (CR) process and the return to the ground state occur rapidly in tens of picoseconds. We noticed that no residual signal was observed in the TA measurements on the nanosecond timescale. This is seemingly inconsistent with the nanosecond fluorescence lifetime in Table 1. We believe this to be associated to a very small fraction of aggregation in THF that is dominating the fluorescence measurements. However, such a small contribution is not detectable in the TA measurements. As a control experiment, the TA spectra of **P1** under protonated conditions were also recorded where the CS processes are blocked and a long-lived ESA is perceived corresponding to the LE state of PMI* (Figure S14b).

Figure 3c shows the TA spectrum of **P2** in toluene excited at 500 nm. When compared to the reference **PMI** (Figure S13), the spectrum contains all the features expected for the perylene excited-state PMI*, including the long-lived GSB (∼520 nm), SE (∼560 nm), and ESA (∼700 nm). The slightly broadened and redshifted ESA of **P2** in toluene likely originates from the inhomogeneous distribution of the molecular conformations and extended π conjugation by the incorporation of *p*-oligophenylene linkers. Interestingly, the long-wavelength absorption of the delocalized excited state at 790 nm in **P1** disappears in **P2**, suggesting that the further separation of **PMI** and **DMA** by an extra phenyl linker limits the oscillator strength of the excited-state absorption that is prominent at a shorter distance. Furthermore, **P2** in THF exhibits an unambiguous CS process (Figure 3c), as supported by the blueshift in ESA caused by **PMI**^•–^ absorption, fast recovery of SE, and an overall shortened lifetime of the ESA suggesting a fast recombination process. Similar to **P1**, the CS process in **P2** is also prohibited by protonating the **DMA** donor. As a result, only the spectral features of the LE state of PMI* and SE are shown upon photoexcitation (Figure S14c).

### Global and Target Analysis

To deconvolute the spectral components and further extract the kinetic rates for the complex excited-state dynamics, global and target analyses were performed with Glotaran.^38^ With this method, the temporal evolutions at all wavelengths were globally fitted to analyze the kinetic profiles (see the SI for details).

**Figure 4:**
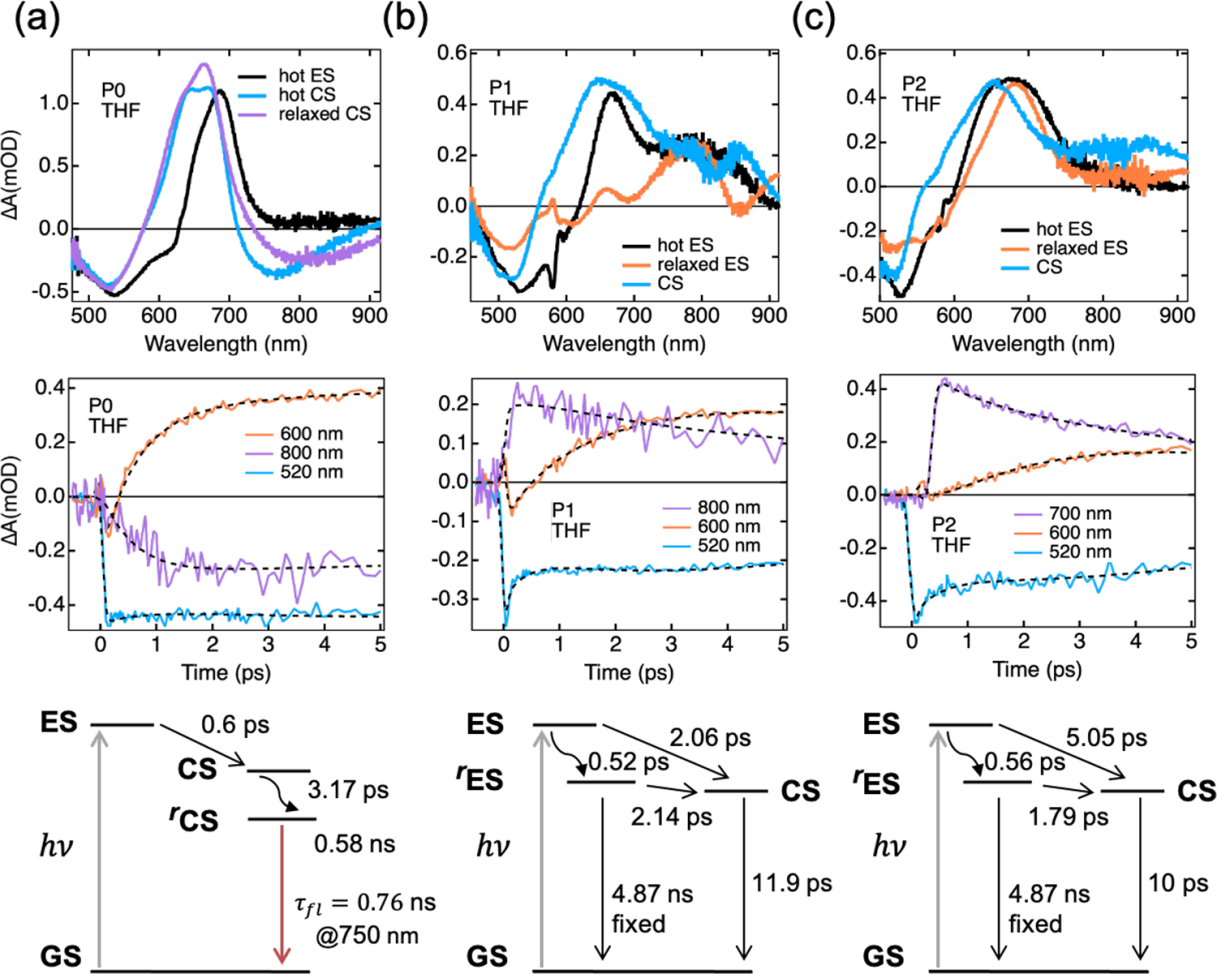
Global (**P0**) and target (**P1/P2**) analysis of femtosecond transient absorption (TA) data for (a) **P0**, (b) **P1**, and (c) **P2** derivatives in THF. First row: EADS (**P0**) and SADS (**P1**/**P2**) obtained from global/target analysis; second row: kinetic profiles of TA data at selected wavelengths and their fits (up to 5 ps); *coherent artifacts affect the kinetics of **P1** and **P2** at initial times (<1 ps). Third row: kinetic scheme for the photodynamics charted with the global and target analysis.

Global analysis with a sequential model is used to analyze the TA spectrum of **P0** in THF. The resulting evolution-associated difference spectra (EADS) are shown in Figure 4a, where three decay components are found, with time constants of 0.6, 3.2, and 580 ps. The first component (0.6 ps) is assigned to the hot excited-state PMI*, as it is formed simultaneously upon excitation and resembles the spectral features of PMI*, analogous to **P0** in hexane (Figure S14a). Following the rapid decay of the hot PMI*, the second component (3.2 ps) displays the characteristics of a CS state, namely, the **PMI**^•–^ absorption at ∼600 nm and the redshifted SE at long wavelengths. The third component (580 ps) retains the spectral features of the second component with a slight difference in the amplitude, suggesting a relaxation process. Thus, we assign the second and third components to be the hot and relaxed CS states, akin to **PMI**^•–^, respectively. Since the charge separation is extremely fast (<1 ps), as indicated by the lifetime of the hot PMI*, it is possible that the initially formed hot CS state (**PMI**^•–^) relaxes in a few picoseconds due to vibrational/solvent reorganization processes.^70,71^ The kinetic traces and global fits of **P0** at three selected wavelengths are shown in Figure 4a, and the strength of global analysis and the reliability of the kinetic rate are justified by the compelling fits (dotted lines) at characteristic wavelengths. As the emission from the CS state grows at 800 nm, the SE at 600 nm is rapidly overshadowed by the rising **PMI**^•–^ absorption within 1 ps, whereas the ground state is still bleached. This means that virtually no PMI* decays back to the ground state while the CS state is formed. The charge recombination time (0.58 ns) obtained from the global analysis is in line with the major component of the fluorescence lifetime recorded at 750 nm (Figure 4a, second row). Computing the CS rate constant (*k*_CS_) for **P0** in moderately polar THF renders a fast rate of ∼1.66 ps^–1^ for the charge separation.

To disentangle the true spectra of the individual excited species and describe the excited-state dynamics in **P1** and **P2**, target analysis with a specific model is used. As demonstrated in Figure 4b,c, the used model considers two pathways for the CS process: (i) direct CS from the hot excited state and (ii) CS from the relaxed excited state. The obtained species-associated difference spectra (SADS) of **P1** and **P2** are presented in Figure 4b,c (first row). Despite the contamination of coherent artifacts at ∼580 nm (at <1 ps), the spectral signatures of hot and relaxed excited states of both **P1** and **P2** are well-described by the black and orange SADS, respectively. The first two components of the SADS for **P1/P2** in THF corresponding to the localized and the delocalized states display the GSB, ESA, and SE signatures analogous to the excited state seen in toluene (Figure S15). The third SADS component corresponding to the nonradiative CS state in both the compounds is represented by the blue curves, representing a **PMI** radical anion (**PMI**^•–^).

To further illustrate the fate of the excited state (ES) and CS states in **P1** and **P2**, kinetic traces and their fits from target analysis at three selected wavelengths are shown in Figures 4b,c (second row). Despite the overlap between the absorption bands of the PMI* and **PMI**^•–^, the hole transfer from PMI* to the **DMA** donor is evident from the increasing characteristic absorption of **PMI**^•–^ at 600 nm and the concurrently decreasing excited-state absorption of PMI* at longer wavelengths. Meanwhile, the slight decrease in the GSB at 520 nm is indicative of the fast charge recombination. Notably, although the donor–acceptor distance is longer in **P2**, there is a striking similarity between the CS rates in **P1** and **P2**. This similarity is further illustrated in Figure 4b,c (third row), wherein a similar kinetic model for the target analysis is depicted for **P1** and **P2**. Alternative models were assessed and then discarded based on the quality of the fits and/or physical interpretability of the estimated rate constants and SADS. In the kinetic model shown in Figure 4b,c, the initially formed hot ES branches to the relaxed ES and the CS state with different rates. In addition to the decay to the ground state, the relaxed ES undergoes charge separation. The CS state subsequently undergoes CR and decay to the ground state in several picoseconds. In both models, the decay of the relaxed ES to the ground state was constrained to be equal to the lifetime of the isolated PMI* in THF. As a result, the target analysis yields similar relaxation lifetimes of the hot ES in **P1** (0.52 ps) and **P2** (0.56 ps). Interestingly, the direct charge separation from the hot ES is about 2 times slower in **P2**, whereas the charge separation from the relaxed ES is faster in **P2**. As a result, the overall CS rate constants are determined to be 0.47 and 0.45 ps^–1^ in **P1** and **P2**, respectively (see Figure S16).

**Figure 5:**
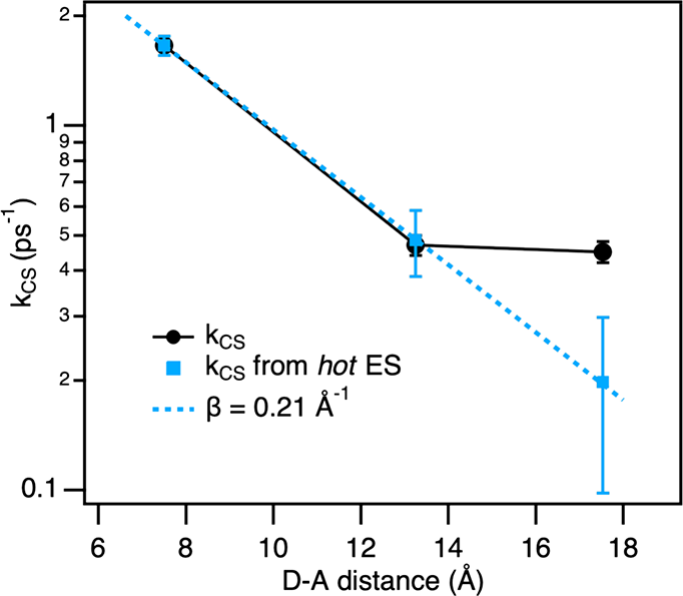
Logarithmic plots of the charge separation rate constants *versus* the donor–acceptor distance for **P0**–**P2**. Black dots represent the overall rate constants, and blue squares represent the rate constants of charge transfer from the hot excited state. The blue line is an exponential fit to the distance dependence with the attenuation parameter β.

Based on the obtained kinetic rate constants, the distance dependence of the CS rate in **P0**–**P2** is plotted on a logarithmic scale in Figure 5. As derived from the target analysis, the formation of the CS state is surprisingly fast in **P2**, resulting in a minuscule attenuation of the overall CS rate from **P1** to **P2** (black dots). According to the coherent tunneling (superexchange) mechanism,^72^ upon elongation of the bridge, the CS rate is expected to drop exponentially with an attenuation factor (β) not less than 0.2 Å^–1^.^73^ On the other hand, the incoherent hopping mechanism^74,75^ is only expected to set in with longer *p*-oligophenylene spacers (*n* > 3).^33,76^ Thus, a shallow distance dependence cannot be ascribed to either of the mechanisms. However, we notice that the direct CS from the hot ES conforms to the exponential distance dependence with a β value of 0.21 Å^–1^ (Figure 5, blue squares). This indicates that the CS from the relaxed ES is responsible for the deviation from the exponential distance dependence for the overall CS rate.

Noticeably, CR rates also show anomalous distance dependence with **P0** (*k*_CR_=1.7 × 10^9^ s^–1^), **P1** (*k*_CR_= 8.4 × 10^10^ s^–1^), and **P2** (*k*_CR_= 1.0 × 10^11^ s^–1^). Indeed, other factors may play a role in the observed anomalous distance dependence such as distance dependence of the outer-sphere reorganization energy (λ_o_).^77^ Derived from the dielectric continuum model by Marcus and Sutin,^13^ λ_o_ is predicted to increase with increasing *R*_DA_. Such an effect could result in a larger electron transfer rate at a longer D–A distance in the inverted regime.^31,78^ Based on the dielectric continuum model, the value of λ_o_ for **P0**–**P2** in THF is estimated to increase moderately from 1.1 to 1.4 eV (Table S6). Given the estimated free energy (Table S5), the charge separation and charge recombination are expected to occur in the normal and inverted regimes, respectively. Therefore, although the distance dependence of λ_o_ may contribute to the anomalous CR rates, it cannot explain the unusual distance dependence of the CS rates. However, we note that it is the CR rate for **P0** that is distinct from **P1** and **P2**, while the CR rates for **P1** and **P2** show a similar distance independence to their CS rates. To rationalize this anomalous CR rate for **P0**, a hint can be taken from its distinct nature of CR through radiative emission. A more comprehensive picture of photophysics for **P0** is provided in the following parts.

### Electronic Structure Calculations

To shed more light on the unexpected distance-dependent kinetic rates, we performed the electronic structure calculations to investigate the nature of the hot and relaxed excited states. With TD-DFT calculations, the hot and relaxed excited states were studied based on the lowest vertical transition corresponding to the optimized geometries of the ground state and the excited state, respectively (see the Supporting Information).

**Figure 6:**
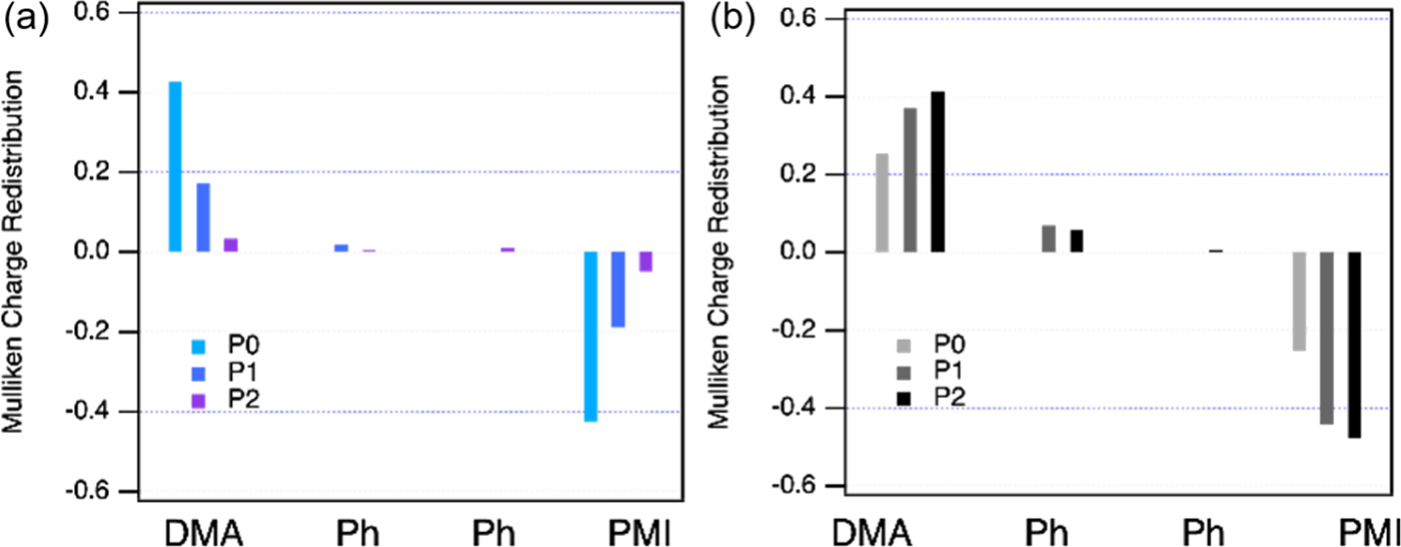
Mulliken charge distribution of the excited state as compared to the ground state for (a) ground-state geometry and (b) excited-state geometry.

It has been previously reported by our group that electron transfer through oligophenylene bridges can be almost distance-independent due to the effect of initial state distribution.^79^ In this case, the vertical excitation of the electron donor involves a direct transition to the bridges.^34^ According to the TD-DFT calculations, the vertical excitation of the **PMI** acceptor in **P0**–**P2** reveals a strong distance-dependent CT character as discussed in the earlier section (Figure 2b and Figure S17 (left panel)). The fundamental (vertical) excitation in **P0** involves a HOMO and HOMO-1 to LUMO CT-type transition. The incorporation of *p*-oligophenylene bridges in **P1** and **P2** renders a highly localized HOMO-1 to LUMO transition localized on the perylene core. To quantify the charge delocalization in the hot ES, the difference of Mulliken charge distribution in the excited state with respect to the ground state was summed for the **DMA** donor, the **PMI** acceptor, and each phenyl unit (Figure 6a). In **P0**, the lowest vertical transition leads to substantial charge redistribution over **DMA** and **PMI** units, indicating that CT in **P0** can be triggered instantaneously upon photoexcitation. Such a strong CT character upon vertical excitation is thus postulated to aid the ultrafast CS in **P0** that is seen experimentally.^69^ Subsequently, the degree of partial CT character decreases significantly with an increasing distance among the **PMI** and **DMA** units. As a result, the difference between the Mulliken charges in the lowest vertical excited state and that in the ground state is negligible for **P1** and **P2**, suggesting a highly localized hot excited state.

To substantiate the extent of ES geometric relaxation and its effect on the CS rates, geometries of the relaxed ES in **P0**–**P2** were optimized. Since it is recognized that the conformational effect can play an important role in determining the π conjugation through the system,^80^ we first assessed the conformational changes upon geometry relaxation in the excited state by comparing the dihedral angles between adjacent fragments. As listed in Table S7, the conformation of the relaxed ES has a propensity for more planarity, especially between **PMI** and its adjacent phenyl unit when compared to the hot ES and the ground-state geometries, where they are identically more twisted. The relaxation timescale of ∼0.5 ps found by target analysis is in line with the subpicosecond torsional relaxation leading to delocalized CT character observed in donor−π–acceptor copolymers.^81,82^ Indeed, as a result of the favored planar structure, the HOMO and HOMO-1 charge densities in the relaxed ES of **P0**–**P2** are widely distributed over the entire molecule suggesting substantial charge delocalization (Figure S17, right panel). It is recognized that the conformations optimized from the calculations in vacuum are not the best representative of the conformations in an ensemble of many molecules in solution. Therefore, to demonstrate the trend for planarity in the excited state, the relative potential energy surfaces (PES) of the ground state (S_0_) and the lowest excited state (S_1_) in **P0** along with the solvent stabilization energy were calculated and are plotted in Figure S18. The PES of S_0_ suggests a large degree of rotational freedom in the ground state at room temperature (0.025 eV), varying from 50 to 135^°^. In contrast, the same energy barrier only allows a rotational freedom of ∼10^°^ around the PES minimum (∼45^°^) in S_1_ regardless of the solvent effect. Hence, it is reasonable to expect that randomly distributed conformations in the ground state are mostly replaced by more planar arrangements in the relaxed excited state. Accordingly, the planar relaxed ES exhibits a higher degree of charge delocalization and higher charge transfer character at the longer donor–acceptor distance, as illustrated in Figure 6b. This explains how the relaxed ES exhibits a stronger CT character due to bridge planarization and in turn accelerates the charge separation at the longer distance.

**Figure 7:**
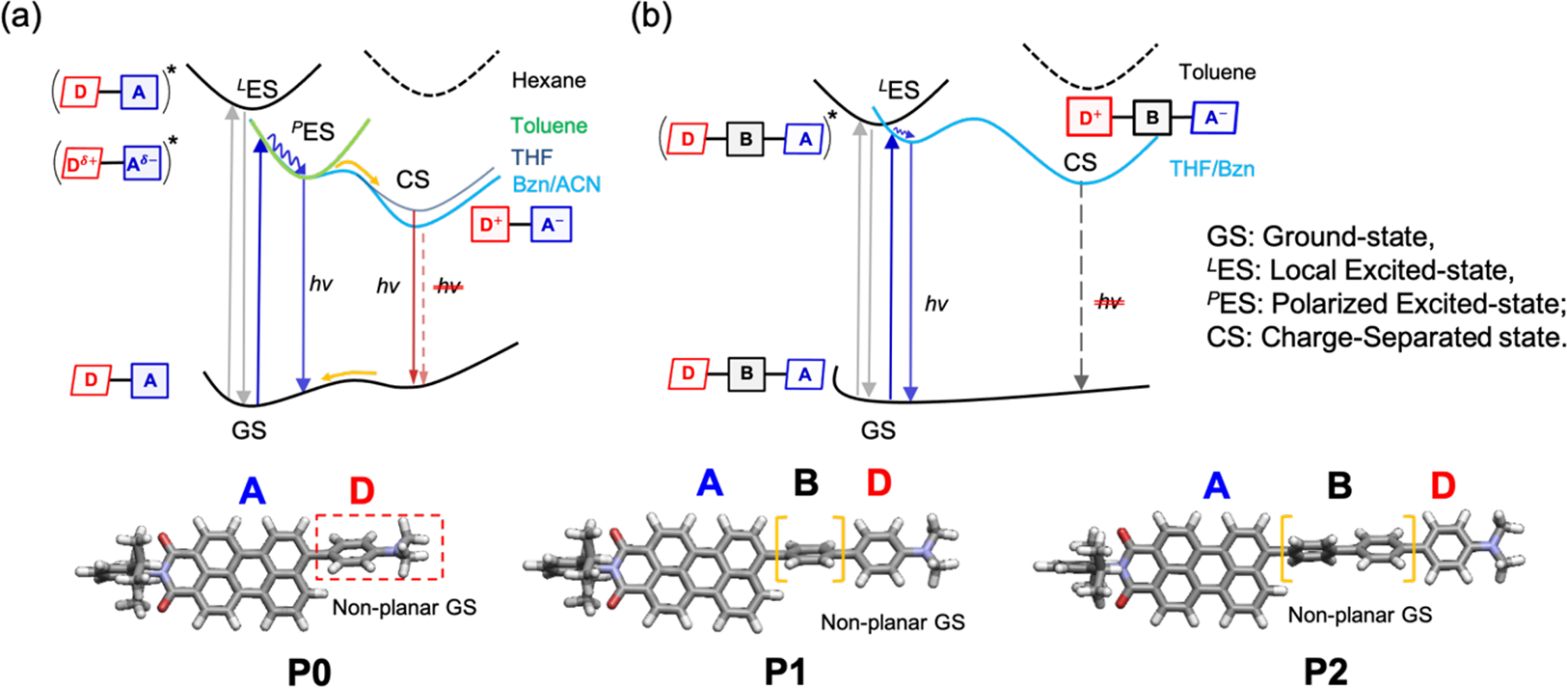
Comprehensive narration of the photoexcited-state processes in (a) **P0** and (b) **P1**/**P2** in nonpolar (highlighted black) and polar solvents (highlighted blue). Geometry-optimized structures (CAM-B3LYP/DZP) of the ground state for **P0**–**P2** are shown for reference.

A careful inspection of the solvent-dependent steady-state spectroscopic results, TD-DFT, TA experiments, and global/target analysis suggests that **P0**–**P2** exhibit a distinct excited-state character as a function of the donor–acceptor distances and solvent polarity. The donor–acceptor separation dictates the ground-state electronic interaction and determines where the initial charge density in the ground state gets redistributed after the FC vertical transition, whereas the solvent polarity commands the subsequent photodynamics from the excited state. For **P0**, the direct coupling between **PMI** and **DMA** renders a strong charge delocalization in the hot ES upon FC vertical excitation. The subsequent de-excitation process now becomes solvent-dependent, and complex photodynamics is obtained depending on the solvent polarity (Figure 7a). Herein, in nonpolar hexane, the relaxed ES is formed with the characteristic of a normal LE state, whereas in weakly polarizing toluene, a polarized relaxed ES is created with a redshifted CT-like emission and a substantial Stokes shift (see Figures 2a and 7a, Figure S8, and Table 1). In moderately polar THF, **P0** exhibits dual emission characteristics owing to the access to at least two different emitting states: (i) a (CT) polarized ES (μ_e_ = 21.24 D) and (ii) CS state (μ_e_ = 30.04 D) (Figure 7a and Table S4). Since the point dipole approximation estimates 1 D = 0.208 eÅ, μ_e_ values of 21.24 and 30.04 D over a distance of 8.93 *Å* for **P0** translate to a moderate (0.49 e) and a fractional degree of charge transfer (0.70 e) for the polarized ES and the CS states, respectively.^58,69,83^ The emissive nature of the CS state in THF suggests that the charge separation is fractional here, and perhaps, a remarkable degree of charge separation can occur in a more polar solvent.^69^ This is further confirmed by the gradual quenching of the redshifted emission from the CS state in more polar solvents such as Bzn and DMSO (Figure S8).^69,83^ Thus, **P0** is a remarkable molecule that due to the charge delocalization in the ground state has the propensity to populate a (non)polarized excited state depending on the solvent polarity. This is evident as a creation of (i) a nonpolarized (local) ES in nonpolar hexane, (ii) a polarized ES in weakly polar toluene, (iii) a polarized ES and CS state (fractional CS) in moderately polar THF, and (iv) a polarized ES and CS state (near-quantitative CS) in highly polar solvents (Figure 7a).^69^ The current observation is in line with the seminal reports on the charge separation that happens via an intermediate partial CT state in organic DA chromophores.^69,84−86^ Additionally, the geometric reorganization and the solvatochromic excited-state character in the dyad **P0** can be compared to the twisted intramolecular charge transfer (TICT) chromophores.^64,84,87^

The introduction of the *p*-oligophenylene bridge between the **PMI** and **DMA** units breaks the coupling of D/A chromophores in the ground state for **P1**/**P2** (Figure 7b) as evident from the marginal solvatochromic effect and the limited charge delocalization in the hot ES upon FC vertical excitation. Furthermore, the polar solvent opens the access to a CS state for **P1**/**P2** as confirmed by the presence of the **PMI** radical anion in the TA experiments. Thus, the ultrafast charge separation for **P0** in THF is facilitated by the instantaneous population of an intermediate (CT) polarized excited state upon vertical excitation. Meanwhile, in **P1**/**P2**, the charge separation occurs directly from the hot ES and the relaxed ES (polarized) in polar solvents, albeit with different rates. Herein, not only the nature and the mode of the charge separation vary as a function of the distance in **P0** and **P1/P2**, but also, the extracted kinetic rates also add a new caveat to the classic distance dependence for CS rates.

## Conclusions

Herein, we report a detailed investigation of the factors that adds a new caveat to the classic nature of the distance-dependent charge separation in **PMI**-*p*-oligophenylene-**DMA** (**P0**–**P2**) compounds. A combined experimental and computational investigation of the charge separation photodynamics reveals that deviation from the distance dependence originates from the relative changes in charge delocalization and charge transfer character in the excited state that scales as a function of bridge separation in **P0**–**P2**. At shorter bridge separation (**P0**), a substantial ground-state D/A electronic interaction and charge delocalization in polar solvents render the population of a strong (CT) polarized excited state upon vertical excitation. The creation of a polarized excited state further aids in an ultrafast charge separation as well as exhibits a high solvent-controlled excited-state character for **P0**. The effect of ground-state orbital mixing and the polarization of the excited state gradually fades with the increasing distance between D/A units in **P1**/**P2**. However, the observed CS kinetic rates in **P2** are much faster than anticipated and represent a deviation from the expected exponential distance-dependent attenuation of CS rates in DBA chromophores.

A detailed kinetic analysis suggests two pathways for forming the charge-separated state: one from the initially formed hot excited state and the other one via the relaxed excited state. While the former shows an attenuation factor of β = 0.21 Å^–1^, the latter shows a large deviation leading to an overall shallow distance dependence for charge separation. To understand the origin of the two distance dependences, we further provided compelling theoretical evidence to demonstrate the distinct nature of the initial states for these two charge transfer pathways. Upon increasing the donor–acceptor distances, a significant increase of the charge delocalization is apparent in the relaxed excited state. This leads to a substantial charge transfer character and a fast charge separation from the relaxed excited state in **P2** than anticipated and compensates for the slower charge separation from the highly localized hot excited state. These findings highlight that the exponential expression for describing the distance dependence at a short DA distance should be used with caution as the charge delocalization along the π-conjugated systems may play an important role in determining the charge separation rates.

## Supporting Information Available

Experimental methods, complete details on the syntheses, cyclic voltammetry, photophysical characterization, TA measurements, global/target analysis, theoretical calculations, and ^1^H and ^13^C NMR spectra of all synthesized compounds (PDF)
