# Supplementary material for: Fast Charge Separation in Distant Donor–Acceptor Dyads Driven by Relaxation of a Hot Excited State
Source: J Phys Chem C Nanomater Interfaces. 2022 Oct 20;126(45):19250–61. doi: 10.1021/acs.jpcc.2c05754 (PMC9677426; doi:10.1021/acs.jpcc.2c05754)
Supplement: Supplementary file 1 — jp2c05754_si_001.pdf [file jp2c05754_si_001.pdf]

Supporting Information for

Fast Charge Separation in Distant Donor-Acceptor Dyads Driven by Relaxation of Hot Excited State

Zimu Wei,‡ Abbey M. Philip,‡ Wolter F. Jager* and Ferdinand C. Grozema**

Department of Chemical Engineering, Delft University of Technology, Van der Maasweg 9, 2629 HZ Delft, The Netherlands

Table of Contents

1. Experimental Section S2
   - **A. Materials** S2
   - **B. Methods** S2
2. Syntheses S4
3. Cyclic Voltammetry S8
4. Photophysical Properties of the Model and DBA Compounds S10
5. Molecular Simulation for the Ground-State Structures S14
6. Distance-Dependent Protonation S16
7. Rehm-Weller Analyses[19-21] S18
9. Transient Absorption Spectra S19
10. Excited-State DFT Calculations S21
11. NMR Spectra S22
12. References S29

1. Experimental Section

A. Materials

The compounds **1** (**PMI**),[1] **2**,[2] and **3**,[3, 4] were synthesized according to the previously described procedures. All other reagents utilized in the syntheses were used as received from the manufacturers, unless otherwise stated. The purification of the products was performed by column chromatography. The TLC plates and the sorbent for the column chromatography (silica gel 40−63, mesh size 0.230−0.400 mm) were purchased from commercial suppliers.

B. Methods

NMR The NMR spectra were recorded with 400 MHz pulsed Fourier transform NMR spectrometer in $CDCl_3$ at room temperature. The chemical shift values are given in ppm and J values in Hz. Mass analysis of **P0-P2** were performed on a Thermo Scientific LTQ XL Linear Ion Trap Mass Spectrometer. The dilute samples (micro to nanomolar concentration) were prepared in analytical grade acetonitrile and analyzed in positive ionization mode using an electron spray ionization (ESI) method.

Electrochemical Analysis. The electrochemical behavior of the compounds was studied by cyclic voltammetry (CHI 600D electrochemical analyzer) in a three-electrode single-compartment cell consisting of a platinum electrode as the working electrode, a Ag/AgCl wire as the reference electrode, and a Pt wire as the counter electrode (scan rate = 50 mV/s). The cell was connected to the computer controlled potentiostat (CH Instruments Inc. 600D). A predried mixture of chloroform:acetonitrile (2:3) containing 0.1 M tetrabutylammonium hexafluorophosphate was used as solvent. The samples were degassed via nitrogen purging prior to the experiment and the concentration of the prepared samples were ca. 0.5 mM. Under these experimental conditions, the oxidation potential for ferrocene (external reference) was observed at 0.43 V (*vs.* Ag/AgCl).

Optical Spectroscopy. Absorption measurements were performed in PerkinElmer Lambda 40 UV−vis spectro-photometer. Photoluminescence studies were done in Horiba Jobin Yvon SPEX Fluorolog 3 spectrophotometer. For quantum yield measurements, the formula for optically dilute solutions (A ≤ 0.10) was used.[5] Fluorescence quantum yields of the **PMI** and DBA compounds (**P0**, **P1**, **P2**) were determined by using N,N´-bis(1-hexylheptyl)-perylene-3,4,9,10-tetracarboxy bisimide (ϕ_F = 0.99 in $CHCl_3$) as a reference.[6,7] The UV-vis absorption and emission experiments under protonated condition were carried out using 48% HBr (in water) as the proton source. A small amount of the acid (2-2.5 microliter) was added to the solution of

the DBA samples in THF/Bzn. Fluorescence lifetimes were recorded on an Edinburgh LifeSpec-ps spectrometer with a fixed excitation wavelength of 404 nm.

Femtosecond transient absorption spectroscopy. Pump-probe fs-TA measurements were performed on a tunable laser system using a YB-KGW oscillator (Light Conversion, Pharos SP-06-200). The fundamental laser pulse (1028 nm) was operating at a frequency of 5 KHz (2.5 KHz repetition rate) with a pulse duration of 180 fs. The pump light was generated by sending the fundamental beam through an optical parametric amplifier (Light Conversion, ORPHEUS-PO15F5HNP1), performing nonlinear frequency mixing and producing an output beam with tunable wavelengths (310-1330 nm). The probe light was obtained by focusing a small fraction of the fundamental beam in a sapphire crystal, producing a broadband continuum spectrum (450 -915 nm). By varying the time delay between the pump pulse and probe pulse, the change in the absorption spectrum was recorded as a function of time using a commercial TA spectrometer (HELIOS, Ultrafast Systems). The resulting two-dimensional difference absorption spectrum, ΔA, was acquired as $\Delta A = \log(I_{pump\text{-}on}/I_{pump\text{-}off})$.[8] All spectra were corrected for the chirp of the white probe light and the polarization effect due to the diffusive reorientation. To assure the stability of the pump pulse, the deviation of the pump power before and after each measurement was smaller than 10%. All compounds were measured in a quartz cuvette with 2 mm path length under continuously stirring.

Global and target analysis. The two-dimensional TA data were analyzed by global and target analysis using Glotaran.[9] With this method, the 2D data matrix, $\Psi(\lambda, t)$, is modeled as linear combinations of n components given by the equation:

$$\Psi(\lambda, t) = \sum_{l=1}^{n_{comp}} c_l(t)\varepsilon_l(\lambda)$$

Each component has its own spectrum, $\varepsilon_l(\lambda)$, that following a certain concentration profile, $c_l(t)$. To be specific, with global analysis a sequential kinetic model is used to describe the evolution of one component into the other with increasing time constants. In addition to the rate constants, each component is characterized by its own evolution associated difference spectrum (EADS). Although the EADS may reflect mixtures of excited species due to the simplicity of the sequential model, the global analysis does provide important information on the temporal evolution of the system. For more complex systems, that following non-sequential kinetics, the target analysis with a specific kinetic model should be used. The resulting species associated difference spectra (SADS) represents the true spectra of the individual excited species based on the used kinetic model.

Theoretical calculations.

Relative potential energy surface (PES) of S_0 in **P0** was calculated by fixing the dihedral angle between the **PMI** and **DMA**, while leaving the rest of the geometry to optimize freely. Based on the PES of S_0, the PES of S_1 in **P0** was then built up by adding the lowest vertical transition energy for the corresponding ground state geometry. To demonstrate the effect of solvent stabilization by THF on the PES of S_1, the Conductor like Screening Model (COSMO)[10] was used as the solvation method. For a given geometry, the single-determinant states were specified by setting the corresponding electron occupation numbers explicitly. The solvent stabilization energy of a specific state was then calculated by subtracting its total bonding energy calculated in THF from that calculated in vacuum. Therefore, the solvent stabilization energy for the ground state S_0 was directly obtained by calculating the difference between the total bonding energy in THF and that in vacuum based on single point calculations. For the first excited state S_1, the overall solvent stabilization energy was calculated as a linear combinations of stabilization energies for the dominant determinates that making up the excited state.

2. Syntheses

The syntheses scheme and the structure of the reference **PMI** and the DBA derivatives are depicted in Figure 1b and the detailed syntheses procedure and characterization is provided below. The synthesis of **P0** involves a one-step reaction of compound 9-bromo **PMI** (**2**) with 4-(N,N-dimethylamino)phenylboronic acid pinacol ester under Suzuki-Miyaura reaction condition. For compounds **P1** and **P2**, the synthesis of intermediate **P1Br** and **P2Br** was performed via Suzuki-Miyaura coupling of compound **3** with 1-bromo-4-iodobenzene and 4-bromo-4'-iodobiphenyl, respectively. Intermediates **P1Br** and **P2Br** were subsequently cross-coupled with 4-(N,N-dimethylamino)phenylboronic acid pinacol ester to provide **P1** and **P2** derivatives in reasonable yields.

Synthesis of P0:

In a 25 ml round-bottom flask, **2** (0.035 g, 0.0626 mmol), 4-(N,N-dimethylamino)phenylboronic acid pinacol ester (0.031 g, 0.125 mmol), 2 M K_2CO_3 (0.125 mL) and tetrahydrofuran (5 ml) were charged under nitrogen flow at room temperature. The reaction mixture was degassed by purging nitrogen for 20 minutes. Subsequently, $Pd(PPh_3)_4$ was added to the reaction mixture and the reaction vessel was sealed under nitrogen. The reaction mixture was heated to 65 °C and stirred for 24 h. The complete consumption of the

starting material was monitored via TLC analysis. After 24 hours, the heating was stopped and the reaction mixture was cooled to room temperature. The solvent was evaporated and the reaction mixture was extracted with water/dichloromethane (DCM) mixture. The organic layer was dried over anhydrous sodium sulfate and evaporated under vacuum. The dried residue was subsequently chromatographed over silica-60 using DCM:Pet. Ether (1:1) as eluent, to afford the pure product **P0** as a dark purple solid (28.2 mg, 75 %).

^{1}H NMR (400 MHz, $CDCl_3$): δ = 8.67 (d, *J* = 8.0 Hz, 2H), 8.56 – 8.45 (m, 4H), 8.17 (d, *J* = 8.4 Hz, 1H), 7.61 (m, 2H), 7.51 – 7.43 (m, 3H), 7.34 (d, *J* = 7.7 Hz, 2H), 6.90 (d, *J* = 8.1 Hz, 2H), 3.08 (s, 6H), 2.78 (m, 2H), 1.18 (d, *J* = 6.8 Hz, 12H) ppm. **^{13}C NMR (100 MHz, $CDCl_3$)** δ = 164.1, 150.2, 145.7, 144.0, 138.01, 137.9, 132. 8, 132.1, 132.0, 131.1, 130.9, 130.6, 130.0, 129.4, 129.3, 128.5, 128.0, 127.5, 127.4, 126.9, 126.7, 124.0, 123.9, 123.89, 120.7, 120.4, 120.1, 119.7, 112.2, 40.5, 29.1, 24.0 ppm.

MS (ESI-MS): $[M+H]^+$ Calculated for $C_{43}H_{36}N_2O_2$: 601.29; found: 601.50

Synthesis of P1Br:

In a 25 ml round-bottom flask, 1-bromo-4-iodobenzene (0.025 g, 0.0897 mmol), **3** (0.060g, 0.0987 mmol), 2 M K_2CO_3 (0.2 ml) and toluene:ethanol (4:1) mixture (5 ml) were charged under nitrogen flow at room temperature. The reaction mixture was degassed by purging nitrogen for 20 minutes. Subsequently, $Pd(PPh_3)_4$ was added to the reaction mixture and the reaction vessel was sealed under nitrogen. The reaction mixture was heated to 70 °C and stirred for 24 h. The complete consumption of the starting material was monitored via TLC analysis. After 24 hours, the heating was stopped and the reaction mixture was cooled to room temperature. The solvent was evaporated and the reaction mixture was extracted with water/dichloromethane (DCM) mixture. The organic layer was dried over anhydrous sodium sulfate and evaporated under vacuum. The dried residue was subsequently chromatographed over silica-60 using DCM:Pet. Ether (1:1) as eluent, to afford the pure product **P1Br** as a red solid (45.0 mg, 67 %).

^{1}H NMR (400 MHz, $CDCl_3$): δ = 8.66 (d, *J* = 8.0 Hz, 2H), 8.52 – 8.45 (m, 4H), 7.95 (d, *J* = 8.4 Hz, 1H), 7.69 (d, *J* = 8.3 Hz, 2H), 7.64 – 7.55 (m, 2H), 7.51 – 7.45 (m, 1H), 7.43 (d, *J* = 8.3 Hz, 2H), 7.34 (d, *J* = 7.8 Hz, 2H), 2.78 (m, 2H), 1.19 (d, *J* = 6.8 Hz, 12H) ppm. **^{13}C NMR (100 MHz, $CDCl_3$)** δ = 164.0, 145.7, 141.9, 138.7, 137.6, 137.3, 132.4, 132.1, 131.8, 131.6, 131.0,

130.5, 129.5, 129.4, 128.9, 128.9, 128.4, 128.2, 127.2, 126.9, 124.0, 124.0, 123.4, 122.3, 121.0, 120.4, 120.2, 29.1, 24.0 ppm.

MS (ESI-MS): $[M+H]^+$ Calculated for $C_{40}H_{31}BrNO_2$: 636.15, 638.15; found: 636.25, 638.33

Synthesis of P1:

In a 25 ml round-bottom flask, **P1Br** (0.038 g, 0.0597 mmol), 4-(N,N-dimethylamino)phenylboronic acid pinacol ester (0.032 g, 0.131 mmol), 2 M K_2CO_3 (0.12 ml) and THF (12 ml) were charged under nitrogen flow at room temperature. The reaction mixture was degassed by purging nitrogen for 20 minutes. Subsequently, $Pd(PPh_3)_4$ was added to the reaction mixture and the reaction vessel was sealed under nitrogen. The reaction mixture was heated to 65 °C and stirred for 24 h. The complete consumption of the starting material was monitored via TLC analysis. After 24 hours, the heating was stopped and the reaction mixture was cooled to room temperature. The solvent was evaporated and the reaction mixture was extracted with water/dichloromethane (DCM) mixture. The organic layer was dried over anhydrous sodium sulfate and evaporated under vacuum. The dried residue was subsequently chromatographed over silica-60 using DCM:Pet. Ether (1:1) as eluent, to afford the pure product **P1** as a dark red solid (23.0 mg, 57 %).

^{1}H NMR (400 MHz, $CDCl_3$): δ = 8.67 (d, *J* = 8.0 Hz, 2H), 8.57 – 8.46 (m, 4H), 8.14 (d, *J* = 8.4 Hz, 1H), 7.75 (d, *J* = 8.0 Hz, 2H), 7.68 – 7.57 (m, 6H), 7.48 (t, *J* = 7.8 Hz, 1H), 7.34 (d, *J* = 7.7 Hz, 2H), 6.86 (d, *J* = 8.7 Hz, 2H), 3.04 (s, 6H), 2.79 (m, 2H), 1.19 (d, *J* = 6.8 Hz, 12H) ppm. **^{13}C NMR (100 MHz, $CDCl_3$):** δ = 164.0, 150.2, 132.7, 132.1, 132.1, 131.1, 130.6, 130.4, 129.6, 129.4, 129.3, 128.3, 128.2, 127.7, 127.0, 126.9, 126.3, 124.0, 123.7, 120.7, 120.2, 120.0, 112.8, 40.5, 29.1, 24.0 ppm.

MS (ESI-MS): $[M+H]^+$ Calculated for $C_{48}H_{41}N_2O_2$: 677.32; found: 677.50

Synthesis of P2Br:

In a 25 ml round-bottom flask, 4-bromo-4'-iodobiphenyl (0.032 g, 0.0897 mmol), **3** (0.060g, 0.0987 mmol), 2 M K_2CO_3 (0.2 ml) and toluene:ethanol (4:1) mixture (5 ml) were charged under nitrogen flow at room temperature. The reaction mixture was degassed by purging nitrogen for 20 minutes. Subsequently, $Pd(PPh_3)_4$ was added to the reaction mixture and the reaction vessel was sealed under nitrogen. The reaction mixture was heated to 70 °C and stirred for 24 h. The complete consumption of the starting material was monitored via TLC analysis. After 24 hours, the heating was stopped and the reaction mixture was cooled to room temperature. The solvent was evaporated and the reaction mixture was extracted with

water/dichloromethane (DCM) mixture. The organic layer was dried over anhydrous sodium sulfate and evaporated under vacuum. The dried residue was subsequently chromatographed over silica-60 using DCM:Pet. Ether (1:1) as eluent, to afford the pure product **P1Br** as a red solid (39.0 mg, 61 %).

^{1}H NMR (400 MHz, $CDCl_3$): δ = 8.68 (d, *J* = 7.9 Hz, 2H), 8.58 – 8.48 (m, 4H), 8.08 (d, *J* = 8.5 Hz, 1H), 7.75 (d, *J* = 8.1 Hz, 2H), 7.69 – 7.60 (m, 6H), 7.57 (d, *J* = 8.5 Hz, 2H), 7.48 (t, *J* = 8.0 Hz, 1H), 7.34 (d, *J* = 7.8 Hz, 2H), 2.78 (m, 2H), 1.18 (d, *J* = 6.8 Hz, 12H) ppm. **^{13}C NMR (100 MHz, $CDCl_3$):** δ = 164.0, 145.7, 142.8, 139.7, 139.4, 139.2, 137.7, 137.5, 132.6, 132.1, 131.0, 130.6, 130.5, 129.3, 128.7, 128.7, 128.4, 128.3, 127.1, 126.9, 126.1, 124.0, 123.5, 122.0, 121.0, 120.9, 120.3, 120.1, 29.1, 24.0.

MS (ESI-MS): $[M+H]^+$ Calculated for $C_{46}H_{35}BrNO_2$, 712.19, 714.18; found: 712.33, 714.33

Synthesis of P2:

In a 25 ml round-bottom flask, **P2Br** (0.05 g, 0.070 mmol), 4-(N,N-dimethylamino)phenylboronic acid pinacol ester (0.035 g, 0.141 mmol), 2 M Cs_2CO_3 (0.2 ml) and THF (7 ml) were charged under nitrogen flow at room temperature. The reaction mixture was degassed by purging nitrogen for 20 minutes. Subsequently, $Pd(PPh_3)_4$ was added to the reaction mixture and the reaction vessel was sealed under nitrogen. The reaction mixture was heated to 65 °C and stirred for 24 h. The complete consumption of the starting material was monitored via TLC analysis. After 24 hours, the heating was stopped and the reaction mixture was cooled to room temperature. The solvent was evaporated and the reaction mixture was extracted with water/dichloromethane (DCM) mixture. The organic layer was dried over anhydrous sodium sulfate and evaporated under vacuum. The dried residue was subsequently chromatographed over silica-60 using DCM as eluent, to afford the pure product **P2** as a dark red solid (34.5 mg, 65%).

^{1}H NMR (400 MHz, $CDCl_3$): δ = 8.68 (d, *J* = 8.1 Hz, 2H), 8.55 (t, *J* = 8.3 Hz, 2H), 8.50 (dd, *J* = 8.3, 1.9 Hz, 2H), 8.13 (d, *J* = 8.4 Hz, 1H), 7.85 – 7.81 (d, *J* = 8.2 Hz, 2H), 7.79 – 7.62 (m, 8H), 7.61 – 7.57 (m, 2H), 7.52 – 7.45 (m, 1H), 7.34 (d, *J* = 7.8 Hz, 2H), 6.84 (d, *J* = 8.8 Hz, 2H), 3.02 (s, 6H), 2.83 – 2.73 (m, 2H), 1.19 (d, *J* = 6.9 Hz, 12H) ppm. **^{13}C NMR (100 MHz, $CDCl_3$):** δ = 1 164.0, 150.1, 145.7, 143.1, 140.6, 140.5, 138.5, 137.9, 137.8, 137.6, 132.7, 132.1, 132.1, 131.1, 130.6, 130.5, 129.5, 128.5, 128.4, 128.3, 127.6, 127.4, 127.1, 127.0, 127.0, 126.7, 124.0, 123.6, 121.0, 120.3, 120.1, 112.8, 40.5, 29.1, 24.0 ppm.

MS (ESI-MS): $[M+H]^+$ Calculated for $C_{54}H_{45}N_2O_2$: 753.35; found: 753.50

3. Cyclic Voltammetry

Table S1. Electrochemical redox potential (vs. Fc/Fc+) and electronic energy levels (vs. vacuum) of the reference and the DBA derivatives in chloroform:acetonitrile (2:3) mixture.

	E_{ox}^{1} (V)^{a}	E_{ox}^{2} (V)^{a}	E_{red}^{1} (V)^{a}	E_{red}^{2} (V)^{a}	E_{g} (eV)^{c}	E_{LUMO} (eV)^{d}	E_{HOMO} (eV)^{e}
**Dimethyl aniline**	0.365^{b}	---	---	---	3.83	-1.26^{f}	-5.09
**PMI**	0.98	---	-1.377	---	2.33	-3.61	-5.95
**P0**	0.399	0.828	-1.388	---	2.06	-3.61	-5.68
**P1**	0.387^{b}	0.906	-1.376	---	2.18	-3.64	-5.82
**P2**	0.367^{b}	0.902	-1.362	---	2.22	-3.64	-5.85

^{a}scan rate = 50 mVs^{-1} and the potentials are reported as $E_{\frac{1}{2}}$ $(= (E_p^a + E_p^c)/2)$ unless otherwise specified. $^{b}E_p$(peak potential). ^{c}Optical bandgap (E_g=1240/λ_{ae}); where λ_{ae} denotes the absorption edge wavelength in nm. ^{d} E_{LUMO} = $-(E_{red}^{onset}$ + 4.8) eV. ^{e} E_{HOMO} = (E_{LUMO} - E_g) eV; E_{red}^{onset} and E_{ox}^{onset} values are reported in Table S1. $^{f}E_{\mathrm{LUMO}}$ = (E_{HOMO} + E_g) eV.

Table S2. Onset redox potential (vs. Fc/Fc^{+}) of the reference and the DBA derivatives in chloroform:acetonitrile (2:3) mixture.

	E_{ox}^{onset} (V)^{a}	E_{red}^{onset} (V)^{a}
**DMA**	0.286	---
**PMI**	0.859	-1.188
**P0**	0.308	-1.185
**P1**	0.254	-1.162
**P2**	0.240	-1.164

^{a}scan rate = 50 mVs^{-1}.

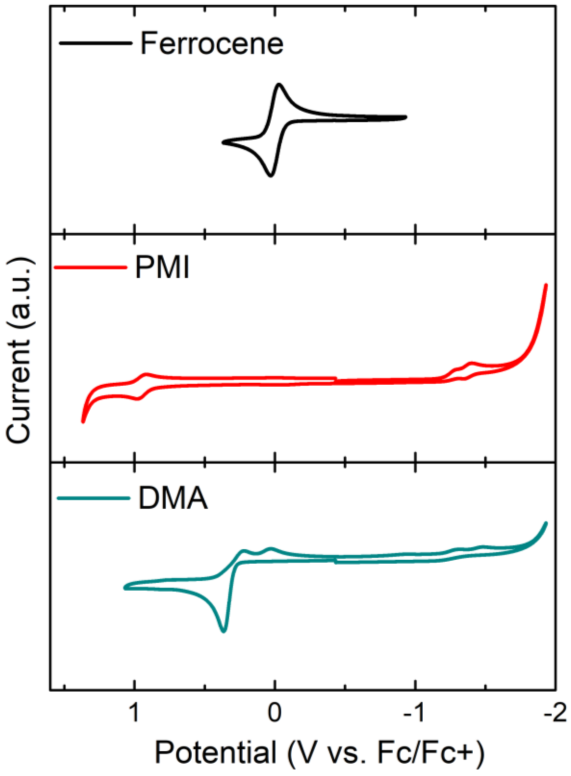

Figure S1. Cyclic voltammograms (vs Fc/Fc^{+}) of the reference compounds in chloroform:acetonitrile (2:3) mixture.

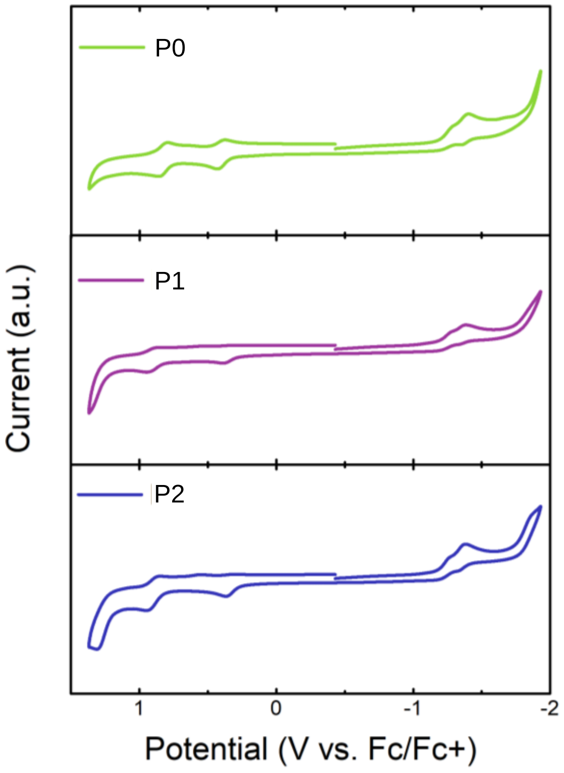

Figure S2. Cyclic voltammograms (vs Fc/Fc^{+}) of the DBA derivatives **P0**, **P1**, and **P2** in

chloroform:acetonitrile (2:3) mixture.

4. Photophysical Properties of the Model and DBA Compounds

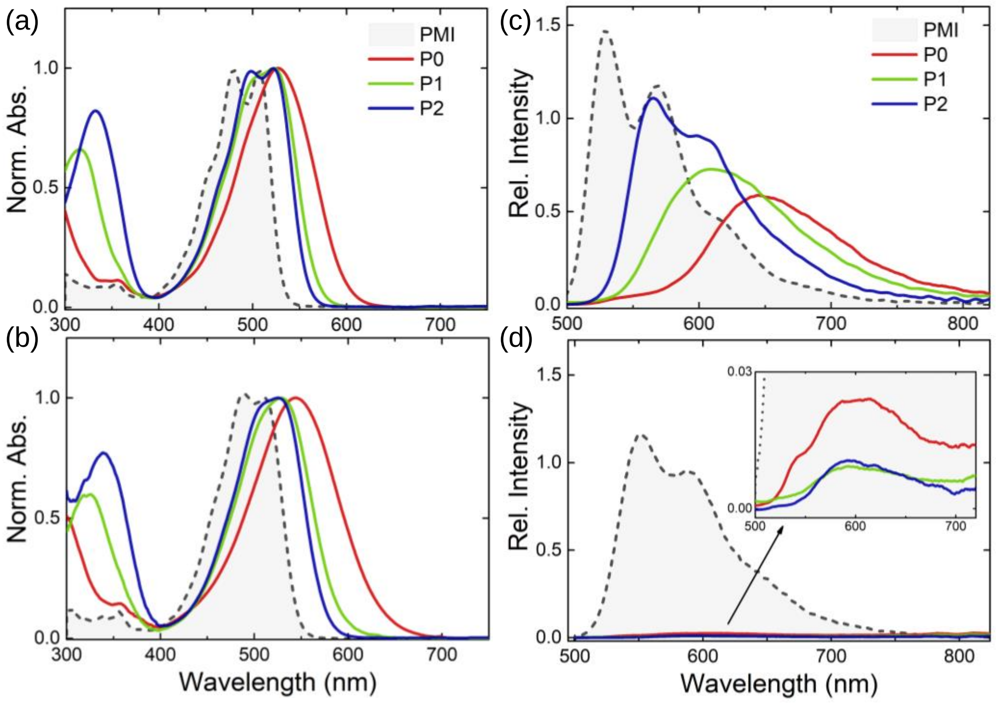

Figure S3. UV-Vis absorption (left) and fluorescence emission (right) spectra of **PMI** and **P0-P2** in toluene (a,b) and Bzn (c, d).

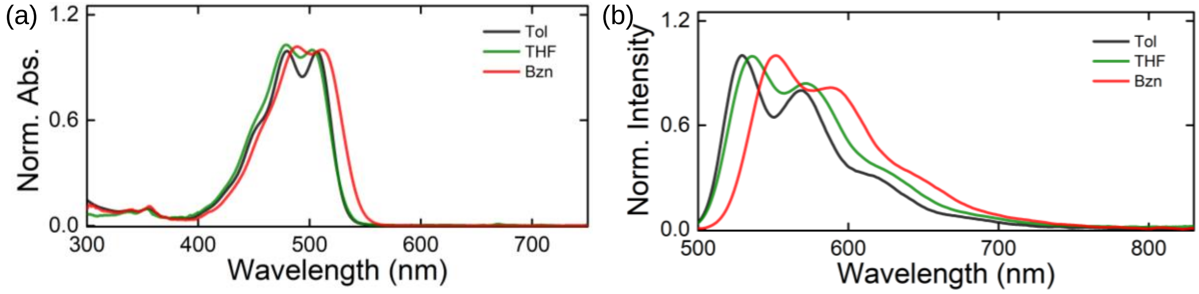

Figure S4. (a) UV-Vis absorption and (b) fluorescence emission spectra of **PMI** in solvents of varying polarity.

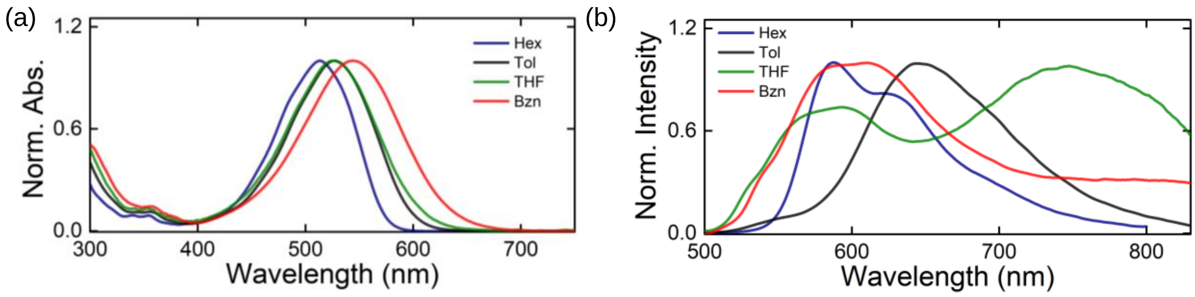

Figure S5. (a) UV-Vis absorption and (b) fluorescence emission spectra of **P0** in solvents of varying polarity.

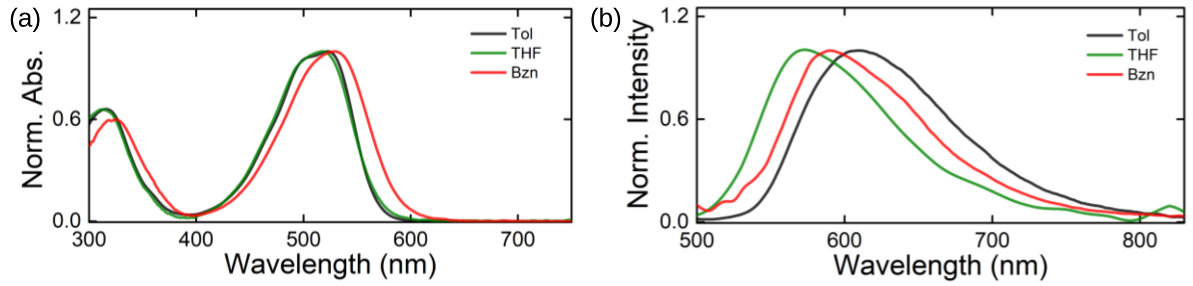

Figure S6. (a) UV-Vis absorption and (b) fluorescence emission spectra of **P1** in solvents of varying polarity.

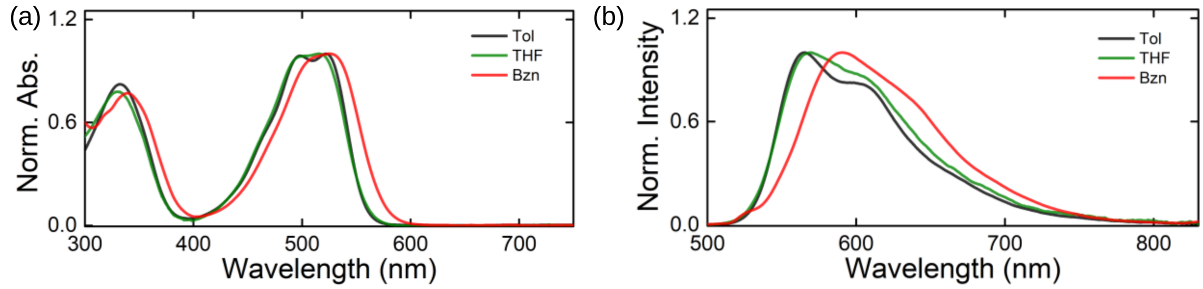

Figure S7. (a) UV-Vis absorption and (b) fluorescence emission spectra of **P2** in solvents of varying polarity.

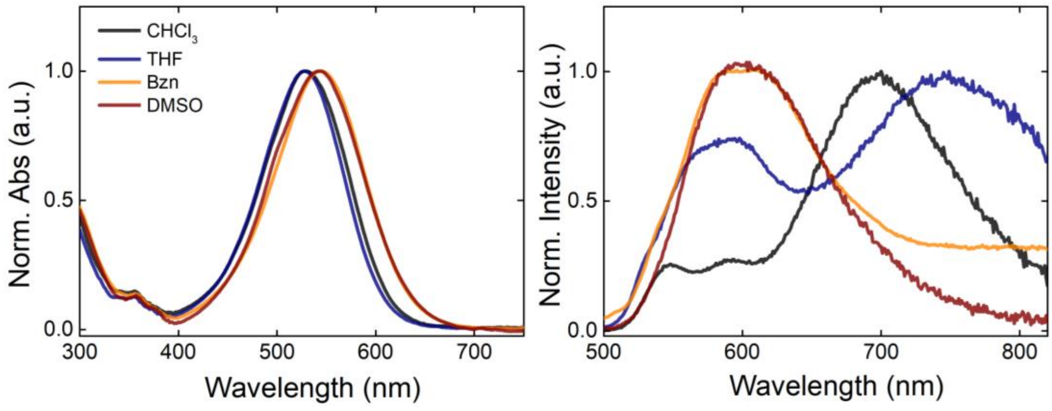

Figure S8. UV-vis absorption (left) and fluorescence emission (right) spectra of **P0** in solvent of increasing polarity ($CHCl_3$ (ε = 4.81); THF (ε = 7.60); Bzn (ε = 25.90); DMSO (ε = 46.70).

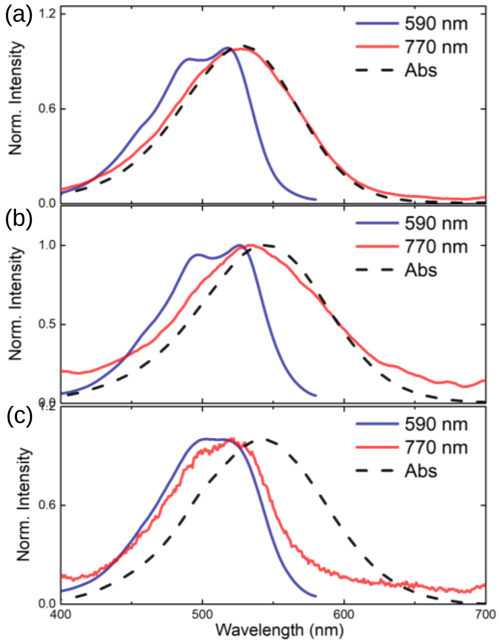

Figure S9. Excitation spectra of **P0** in (a) THF, (b) Bzn and (c) DMSO monitored at the short and long emission wavelengths. For comparison the UV-vis absorption of **P0** (dashed lines) in corresponding solvent is plotted along with the excitation spectra.

Solvatochromic Method for Dipole Moment Calculations[11-13]

Solvent polarity dependent UV-vis absorption and fluorescence emission provide a convenient and facile method to probe the change in the dipole moment between the ground and the excited-state of a fluorophore. Herein, the fluorophore is considered as a spherical point-dipole placed at the centre of a continuous medium with a uniform dielectric medium. The interaction between the fluorophore and the dielectric solvent medium creates a change in the energetics of the ground and the excited-state and as a result the dipole moment associated with them. This approximation neglects the effect of the dispersive interactions caused by the Van de Waals forces and is valid only when the dispersive forces are quite weaker as compared to the dipolar interaction between the fluorophore and the dielectric solvent medium.

The difference in the dipole moment associated with the ground and the excited states can be described as a function of refractive index (n) and the dielectric medium (ε) under consideration.[11-13] To calculate both the ground and the excited state dipole moments, a generalized linear correlation can be utilized[11,13]:

$$\tilde{\nu}_a - \tilde{\nu}_f = m_1 f(\varepsilon, n) + constant \quad (1)$$

$$\tilde{\nu}_a + \tilde{\nu}_f = -m_2 \varphi(\varepsilon, n) + constant \quad (2)$$

$$\text{where } m_1 = \frac{2(\mu_e - \mu_g)^2}{hca^3} \quad (3)$$

$$m_2 = \frac{2(\mu_e^2 - \mu_g^2)^2}{hca^3} \quad (4)$$

Herein, μ_g and μ_e are the ground and excited state dipole moments of the solute; $f(\varepsilon, n)$ is a function of the solvent dielectric constant (ε) and its refractive index (n); $g(n) = \frac{n^2-1}{2n^2+1}$ is a function of the

refractive index; h is Planck's constant; c is the speed of light in vacuum; a is the spherical Onsager cavity radius of the solute molecule.

m_1 and m_2 can be determined from the slopes of the linear plots of $(\tilde{\nu}_a - \tilde{\nu}_f)$ *versus* $f(\varepsilon, n)$ and $(\tilde{\nu}_a + \tilde{\nu}_f)$ *versus* $\varphi(\varepsilon, n)$, respectively. The solvent polarity parameter $f(\varepsilon, n)$ and $\varphi(\varepsilon, n)$ are defined as follows (ignoring the polarizability of solute)[11-13]:

$$f(\varepsilon, n) = \frac{\varepsilon-1}{2\varepsilon+1} - \frac{n^2-1}{2n^2+1} \quad (5)$$

$$\varphi(\varepsilon, n) = f(\varepsilon, n) + 2g(n) \quad (6)$$

$$\varphi(\varepsilon, n) = \frac{\varepsilon-1}{2\varepsilon+1} + \frac{n^2-1}{2n^2+1} \quad (7)$$

Considering that the symmetry of the solute molecule remains unchanged upon an electronic transition, the ground and excited state dipole moments can be calculated from equations 8 and 9[11, 13]:

$$\mu_g = \frac{m_2 - m_1}{2}\left[\frac{hca^3}{2m_1}\right]^{1/2} \quad (8)$$

$$\mu_e = \frac{m_1 + m_2}{2}\left[\frac{hca^3}{2m_1}\right]^{1/2} \quad (9)$$

Dividing equation 9 by equation 8:

$$\mu_e = \frac{m_1 + m_2}{m_2 - m_1}\mu_g; \quad (10)$$

Table S3. Ground and the excited-state dipole moment calculated employing the solvatochromic[11, 13] method.

	**Solvent**	$f(\varepsilon, n)$	$\phi(\varepsilon, n)$	ν_a **(cm^{-1})**	ν_f **(cm^{-1})**	$(\nu_a - \nu_f)$ **(cm^{-1})**	$(\nu_a + \nu_f)$ **(cm^{-1})**	m_1	m_2	μ_g **(D)**	$^a\mu_e$ **(D)**	$^b\mu_e$ **(D)**	$^{c,d}\Delta\mu$ **(D)**
**PMI**	Toluene	0.0132	0.4660	19724	18868	856	38592	2343	3499	1.78	9.02	9.02	7.24
	$CHCl_3$	0.1482	0.5692	19646	18553	1093	38199						
	DCM	0.2171	0.6237	19724	18382	1342	38106						
	THF	0.2096	0.6047	19920	18657	1263	38577						
	Bzn	0.2361	0.7070	19569	18182	1387	37751						
	DMF	0.2744	0.6852	19802	18018	1784	37820						
**P0**	Hexane	0.00027	0.3721	19493	17036	2457	36529	12942	17068	2.92	30.04	21.24	27.48[c], 18.32[d]
	Toluene	0.0132	0.4660	18975	15528	3447	34503						
	$CHCl_3$	0.1482	0.5692	18939	14286	4653	33225						

	THF	0.2096	0.6047	18975	13369	5606	32344						
	Bzn[e]	0.2361	0.7070	18349	12853	5495	31202						
**P1**	Toluene	0.0132	0.4660	19120	16447	2673	35567	7600	9582	2.13	18.47	18.47	16.34
	$CHCl_3$	0.1482	0.5692	19011	17762	1249	36773						
	THF	0.2096	0.6047	19305	17482	1823	36787						
	acetone	0.2842	0.6450	19380	17331	2049	36711						
	Bzn	0.2361	0.7070	18904	16949	1955	35853						
	ACN	0.3054	0.6550	19380	16920	2460	36300						
**P2**	Toluene	0.0132	0.4660	19157	17668	1489	36825	6465	9982	4.12	19.29	19.29	15.17
	$CHCl_3$	0.1482	0.5692	19120	17544	1576	36664						
	THF	0.2096	0.60471	19380	17575	1805	36955						
	Bzn	0.2361	0.7070	19011	16920	2091	35931						
	ACN	0.3054	0.6550	19455	16863	2592	36318						

$^{a}\mu_e = \frac{m_1+m_2}{2}\left[\frac{hca^3}{2m_1}\right]^{1/2}$; $^{b}\mu_e = \frac{m_1+m_2}{m_2-m_1}\mu_g$; $^{c}\Delta\mu = {}^{a}\mu_e - \mu_g$; $^{d}\Delta\mu = {}^{b}\mu_e - \mu_g$. [e]The diffuse red-shifted component was selected as emission band.

5. Molecular Simulation for the Ground-State Structures

Table S4. Dipole moment, excitation energies, oscillator strengths and main contributions to the excited state of the DBA molecules.

Molecule	E_{exc}(eV)	λ (nm)	*f*	Main contributions
**PMI**	2.97	416	0.687	97.5% (H→L)
	4.66	265	0.006	51.3% (H-2→L)<br>42.2% (H→L+1)
**P0**	2.85	434	0.956	81.6% (H→L)<br>15.5% (H-1→L)
	3.60	334	0.004	15.5% (H-1→L)
	4.58	270	0.018	35.4% (H-1→L+1)
**P1**	2.90	427	1.00	84.4% (H-1→L)<br>12.8% (H→L)

	3.71	333	0.013	79.4% (H→L)<br>12.3% (H-1→L)
	4.56	271	0.046	24.4% (H-1→L+2)<br>24.3% (H-4→L)
**P2**	2.91	426	1.03	95.0% (H-1→L)<br>2.2% (H→L)
	3.85	321	0.021	89.97% (H→L)<br>5.3% (H-3→L)
	4.56	272	0.82	28.4% (H→L+4)<br>20.8% (H→L+1)<br>9.9% (H-1→L+1)

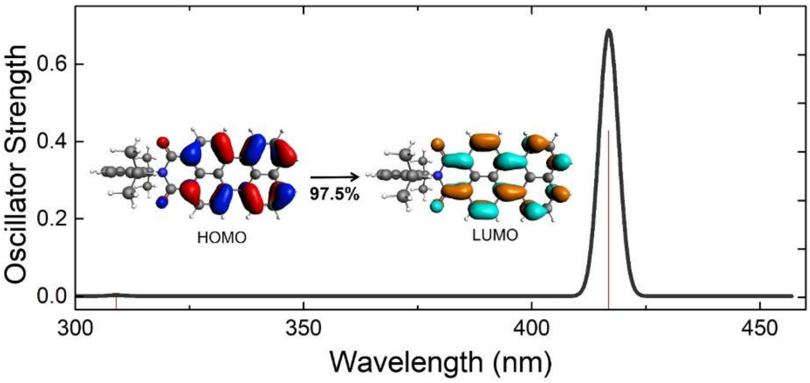

Figure S10. Optical excitation of **PMI** calculated using TD-DFT with DZP/CAM-B3LYP.

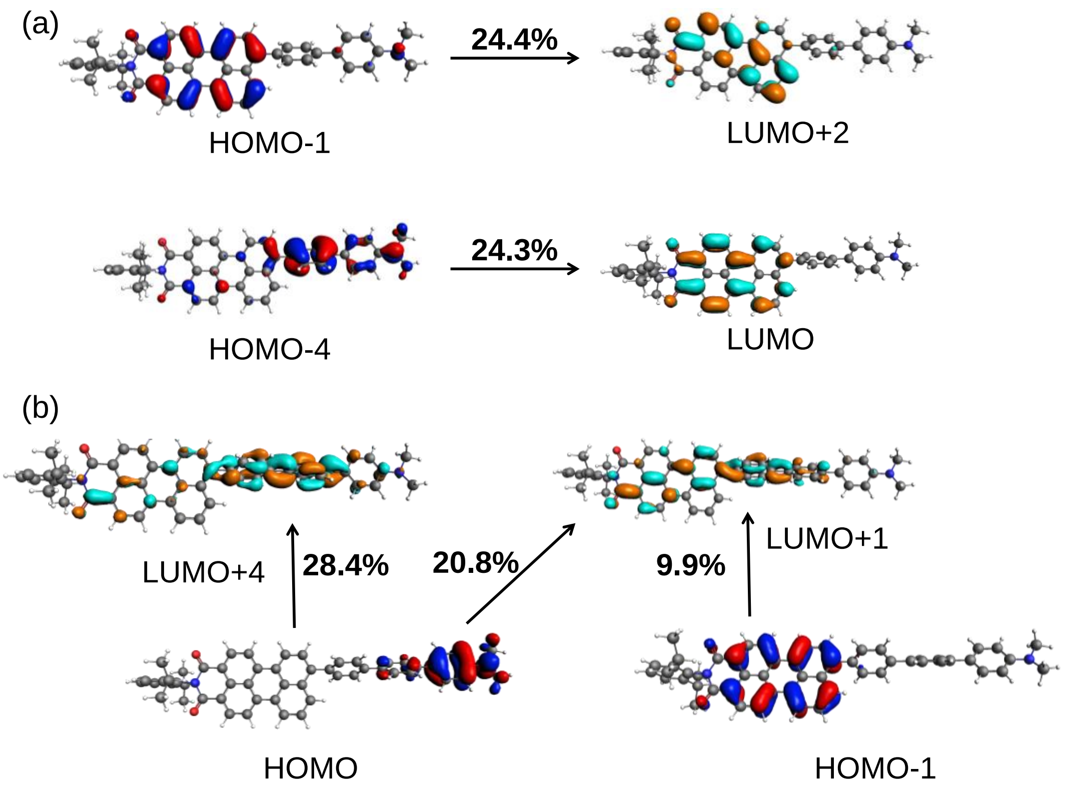

Figure S11. Orbital transitions involved in the high energy absorption band of (a) **P1** (271 nm, $f = 0.046$) and (b) **P2** (272 nm, $f = 0.82$) calculated using TD-DFT with DZP/CAM-B3LYP.

6. Distance-Dependent Protonation

The UV-vis absorption and emission experiments under protonated condition were carried out using 48% HBr as the proton source. A small amount of the acid (2-2.5 microliter) was added to the solution of the DBA samples in THF/Bzn.

The DBA compounds **P0-P2** exhibit a distance-dependent fluorescence emission in weakly polar toluene (Figure S3), whereas a dramatic quenching is observed in polar THF/Bzn (Figure S3) from the different deexcitation processes. To simplify the photoexcited state properties and nullify the complication arising from the CT and CS processes, we attempted protonation of **P0-P2** derivatives in Bzn with hydrobromic acid (HBr, Figures S12). Protonation of the dimethylamino group reduces its electron-donating nature and blocks all the complex CT processes that render a nonradiative deexcitation in polar solvents. The protonation experiments in THF/Bzn reveal a distinct hypsochromic shift in the UV-vis absorption as a function of the distance between **PMI** and **DMA** units for the DBA compounds (Figure S12a, S12c, and S12e). The nature and the magnitude of the hypsochromic shift in the UV-vis absorption of the protonated compounds proves the distance-dependent CT polarization in **P0**-**P2**, as earlier proved by the UV-vis absorption and TD-DFT calculations. Concurrently, the fluorescence

emission for protonated **P0-P2** is synchronously activated from the bright LE state with spectral character similar to unsubstituted **PMI** and a remarkably enhanced quantum yields. The magnitude of the fluorescence enhancement (FE) upon protonation depends on the DBA distances and the solvent polarity (Table 1 (*manuscript*), Figure S12). The FE upon protonation is high (**P1**/**P2**) and moderate (**P0**) when the fluorescence quenching is high and moderate in the neutral state. The red-shift of the emission band in the protonated state reveal a trend of most increased shift in **P2,** then **P1** and the least in **P0**. This observation suggests that the charge delocalization in the excited state of **P2** is more as compared to **P1** and **P0**.

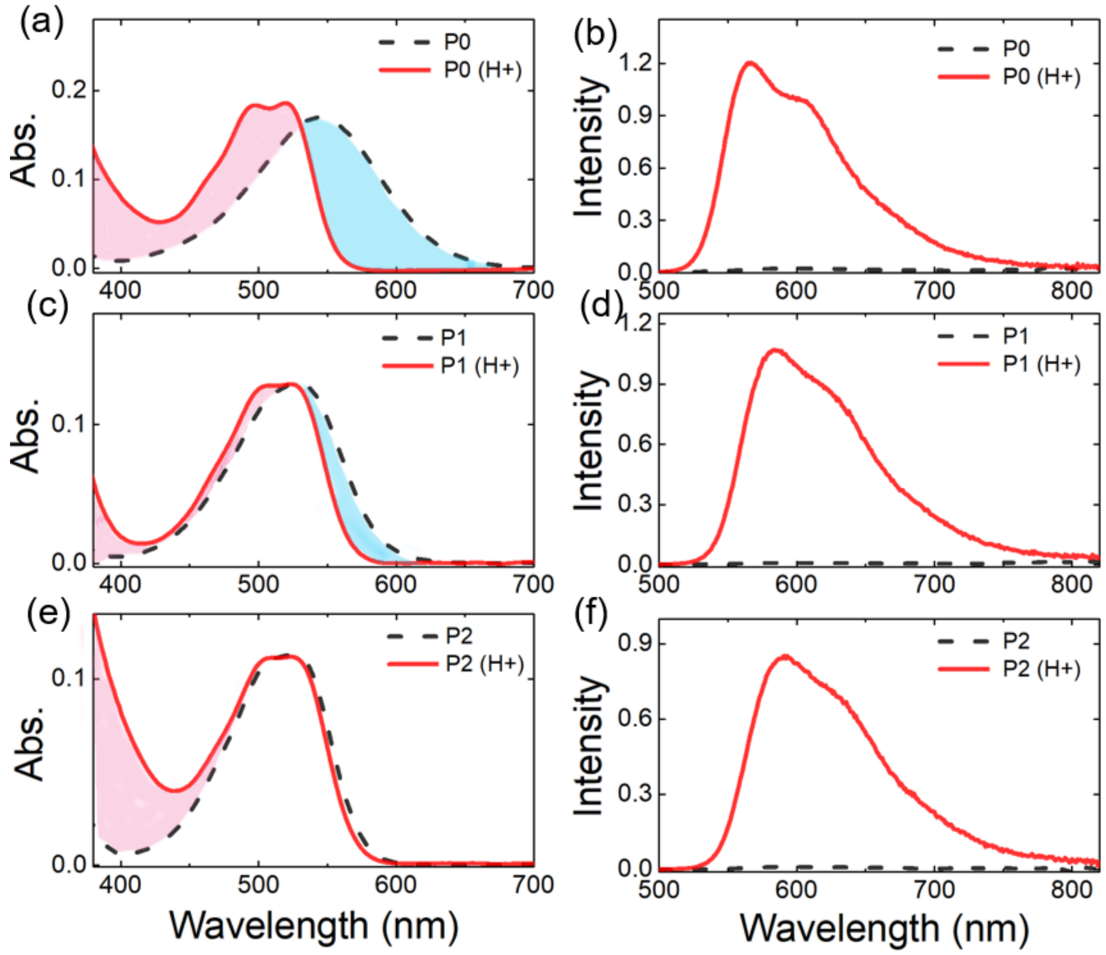

Figure S12. UV-vis absorption (a, c, e) and fluorescence emission (b, d, f) in neutral (dashed black lines) and protonated (bold red line) state for **P0** (1st row); **P1** (2nd row); **P2** (3rd row) in Bzn. Protonation experiments were performed by adding a small amount of HBr (48%) to the solutions containing the DBA compound in Bzn. Shaded area highlight the magnitude of change in UV-vis absorption under protonated and neutral condition.

7. Rehm-Weller Analyses[14-16]

$$\Delta G^{\circ}_{CS} = E_{ox}(D) - E_{\mathrm{red}}(A) - E_{0,0}(D/A) - \Delta G_S \quad (1)$$

$$\Delta G^{\circ}_{CR} = -E_{\mathrm{ox}}(\mathrm{D}) + E_{\mathrm{red}}(\mathrm{A}) + \Delta G_S \quad (2)$$

$$\text{where } \Delta G_S = \frac{e^2}{4\pi\varepsilon_0 r_{\mathrm{DA}}\varepsilon_{\mathrm{S}}} - \frac{e^2}{8\pi\varepsilon_0}\left(\frac{1}{2\,r_{\mathrm{D}}} + \frac{1}{2\,r_{\mathrm{A}}}\right)\left(\frac{1}{\varepsilon_{\mathrm{S}}} - \frac{1}{\varepsilon_{\mathrm{ref}}}\right) \quad (3)$$

In Eq. S1, $E_{ox}(D)$ and $E_{\mathrm{red}}(A)$ are the oxidation potential of the donor and the reduction potential of the acceptor, while and $E_{0,0}(A)$ is the spectroscopic excited state energy of the donor/acceptor. In this equation r_{D} and r_A are the ionic radii of the donor and acceptor radical ions, r_{DA} is the donor-acceptor distance, while $\varepsilon_{\mathrm{ref}}$ and ε_{S} are the dielectric constants of the reference solvent and the chosen solvent for spectroscopy, respectively. Using the Rehm-Weller equation is a rather crude approximation, as it assumes the formation of spherical ions and approaches the charge separation energy by point charges at the center to center distance between the chromophores r_{DA}. The charge separation distance for **P0** (r_{DA} = 8.93 Å), **P1** (r_{DA} = 13.03 Å) and **P2** (r_{DA} = 17.46 Å) were computed from the DFT optimized geometries (CAM-B3LYP/DZP in ADF package). The ionic radius of the perylene acceptor r_A= 6.08 Å, and dimethylaniline (DMA) donor r_D = 2.10 Å was estimated from the DFT optimized geometries employing *volume* function in Gaussian09 Rev. B.01 (B3LYP/6-31g(d,p)). It is obvious that in the apolar solvent toluene, the margins of error in the calculated charge separation energies are significant, whereas the values calculated for Bzn are more accurate. Free energy for charge separation (ΔG°_{CS}) and recombination (ΔG°_{CR}) were calculated using Eq 1 and 2, respectively.[14-16]

Table S5. Rehm-Weller driving force for charge separation (ΔG°_{CS}) and recombination (ΔG°_{CR}) for the DBA derivatives.

	ΔG°_{CS} (Tol)	ΔG°_{CS} (THF)	ΔG°_{CS} (Bzn)	ΔG°_{CR} (Tol)	ΔG°_{CR} (THF)	ΔG°_{CR} (Bzn)
**P0**	0.93	-0.30	-0.65	-3.32	-2.08	-1.68
**P1**	1.22	-0.23	-0.64	-3.61	-2.15	-1.69
**P2**	1.43	-0.13	-0.56	-3.75	-2.17	-1.67

8. Dielectric Continuum Model[17]

To roughly estimate the outer-sphere reorganization energy (λ_o) for **P0-P2** in THF ($\eta = 1.41$, $\epsilon_s = 7.58$), the dielectric continuum model by Marcus was used. In this model, the donor and the acceptor are approximated by two spheres with radii a_1 and a_2:

$$\lambda_o = (\Delta e)^2 \left(\frac{1}{2a_1} + \frac{1}{2a_2} - \frac{1}{r_{DA}}\right)\left(\frac{1}{\epsilon_{op}} - \frac{1}{\epsilon_s}\right) \quad (1)$$

where Δe is the amount of charge transferred, and r_{DA} is the center-to-center distance between the donor and the acceptor. ϵ_{op} and ϵ_s are the optical (the square of the solvent refractive index, η) and static dielectric constants of the solvent, respectively. We used the same values of radii and distance as mentioned above.

Table S6. Estimation of outer-sphere reorganization energy (λ_o).

	λ_o(eV)
**P0**	1.1
**P1**	1.3
**P2**	1.4

9. Transient Absorption Spectra

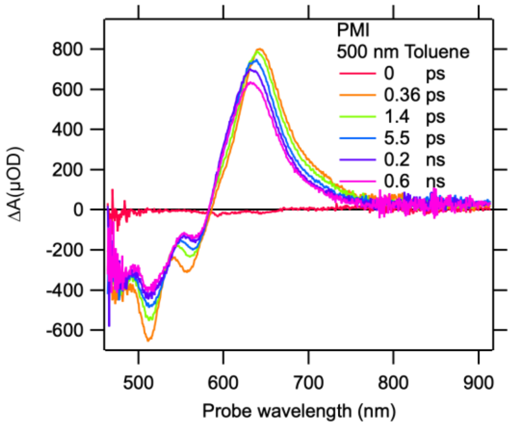

Figure S13. Transient absorption spectra of **PMI** in toluene upon excitation at 500 nm.

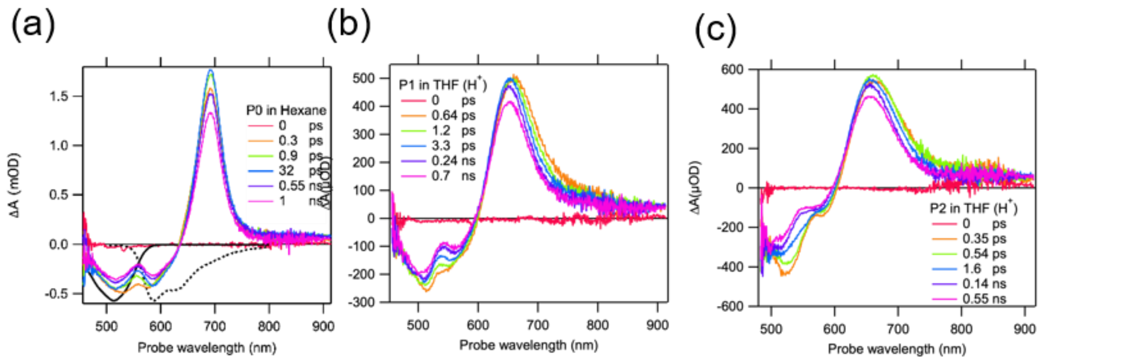

Figure S14. TA spectra of (a) **P0** in hexane upon excitation at 530 nm. TA spectra of protonated (b) **P1** and (c) **P2** in THF upon excitation at 500 nm.

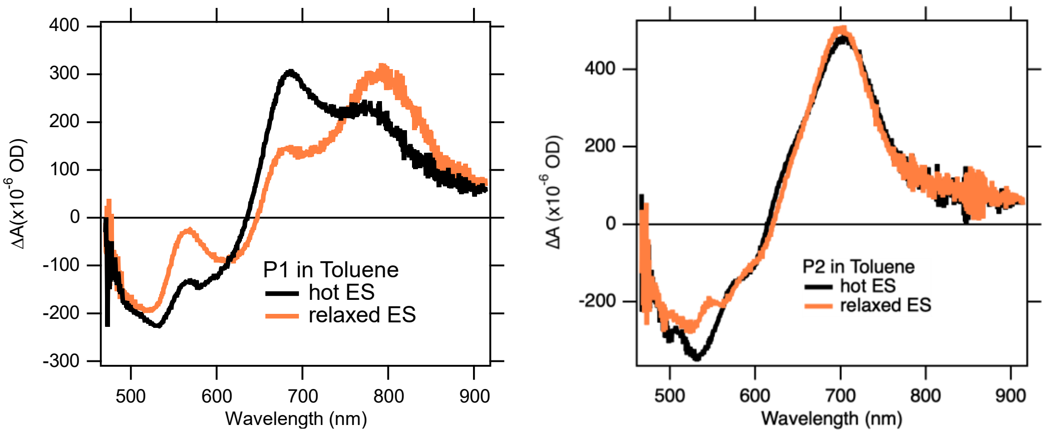

Figure S15. Evolution-associated difference spectra (EADS) of **P1** and **P2** in toluene.

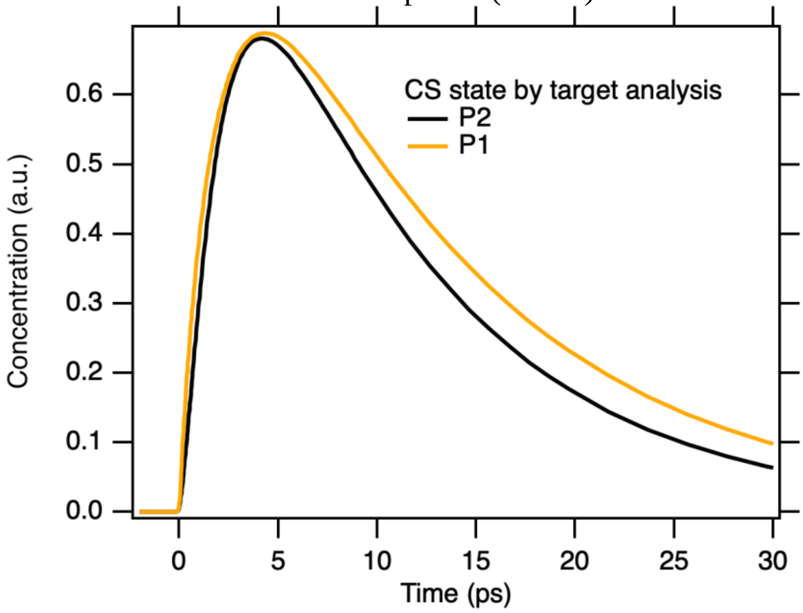

Figure S16. Comparison of the concentration profiles of CS state for **P1** and **P2** in THF.

10. Excited-State DFT Calculations

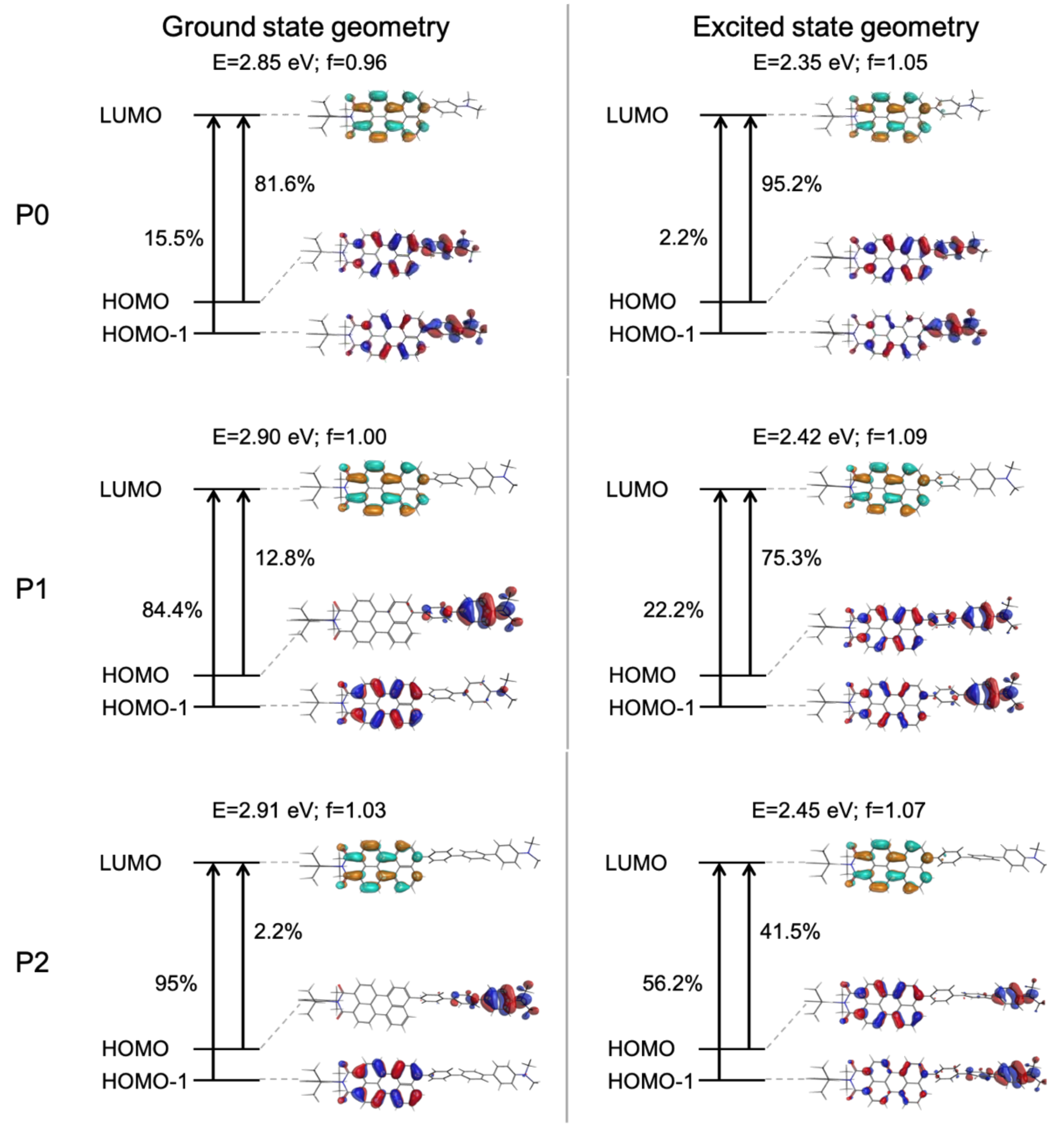

Figure S17. Contributions of single orbital transitions to the lowest vertical excitation in **P0-P2** based on the ground state geometry (left) and excited state geometry (right) calculated using TD-DFT with CAM-B3LYP/DZP. Only the contribution larger than 2% is shown.

Table S7. Dihedral angles in the geometry optimized ground state and excited state.

	Ground state geometry			Excited state geometry		
	Φ_1	Φ_2	Φ_3	Φ_1	Φ_2	Φ_3
P0	63.4°	-	-	45.8°	-	-
P1	69.0°	42.4°	-	51.2°	39.1°	-
P2	71.0°	46.1°	43.5°	55.1°	44.7°	45.3°

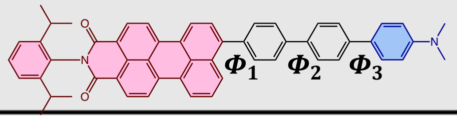

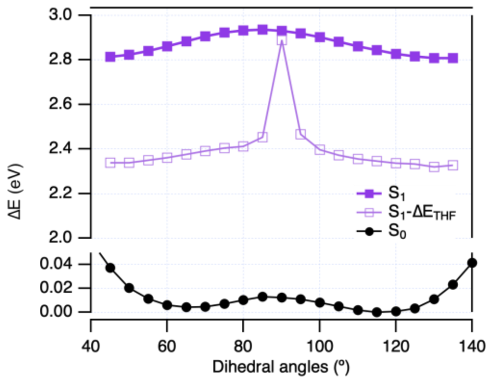

Figure S18. Relative potential energy surfaces (PES) of S_0 and S_1 in vacuum and S_1 in THF as a function of the dihedral angle between **PMI** and **DMA** in **P0** calculated with CAM-B3LYP/DZP. ΔE_{THF} is the amount of energy stabilized by THF in the excited state with respect to the ground state. Note that the PES of the CT state is not shown for the clarity.

11. NMR Spectra

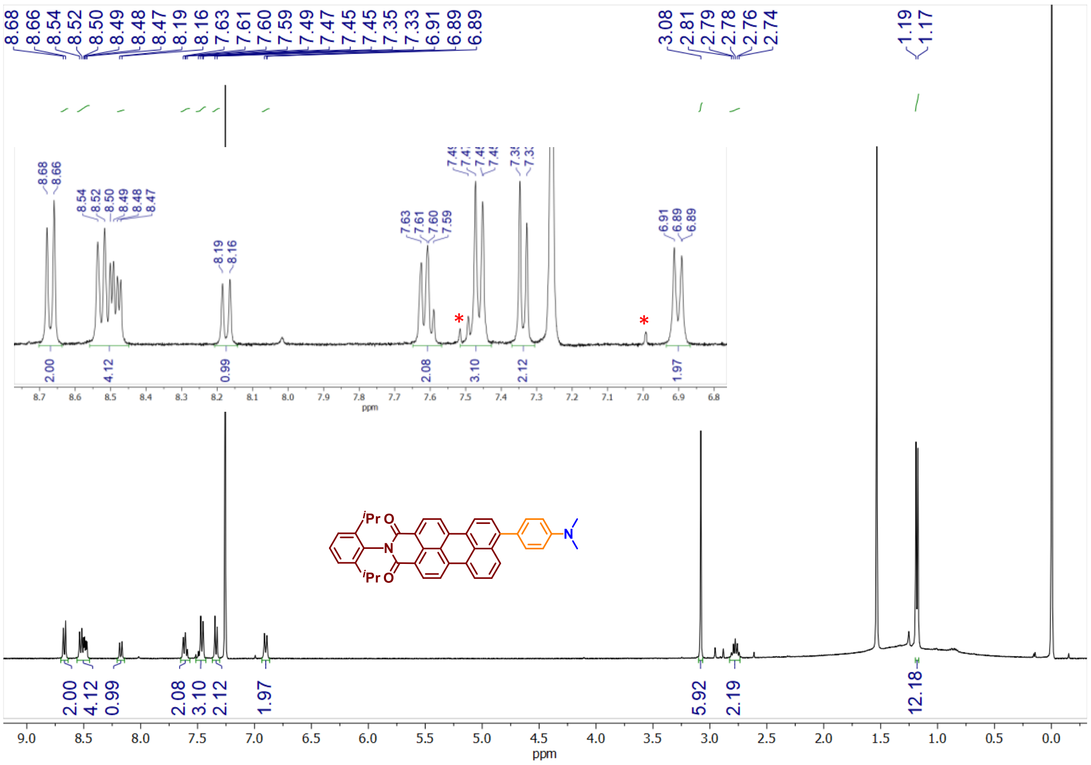

Figure S19. ^{1}H NMR spectrum of **P0** in $CDCl_3$. The asterisks indicate the ^{13}C-satellite peaks arising from the $^1J_{^{13}\mathrm{C-H}}$ (210 Hz) coupling in $CDCl_3$.

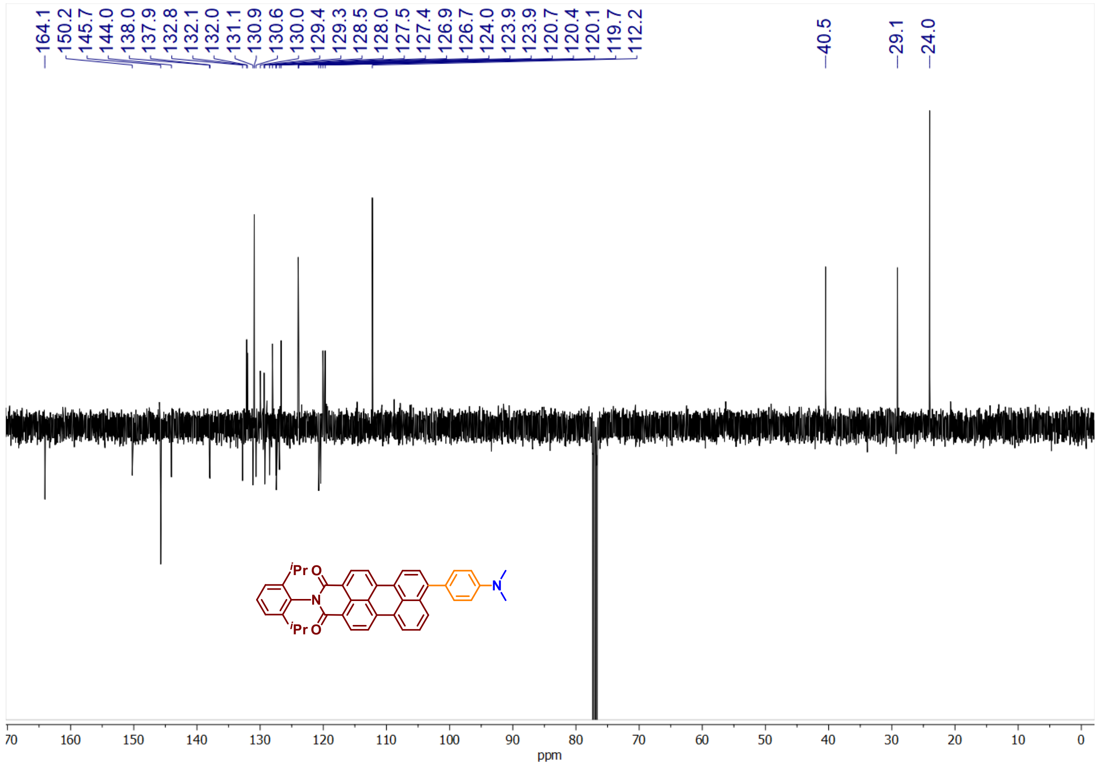

Figure S20. ^{13}C-APT NMR spectrum of **P0** in $CDCl_3$.

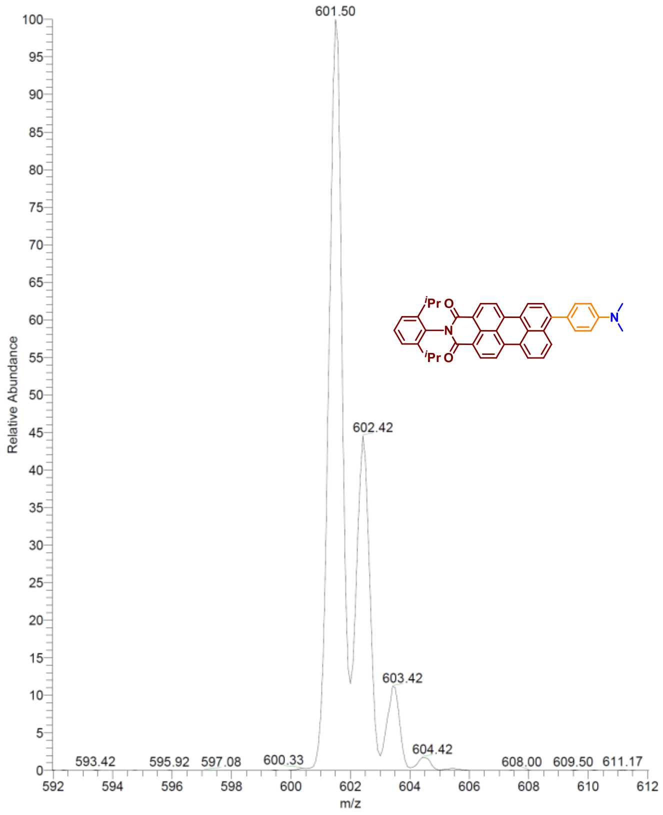

Figure S21. Mass spectrum of **P0**.

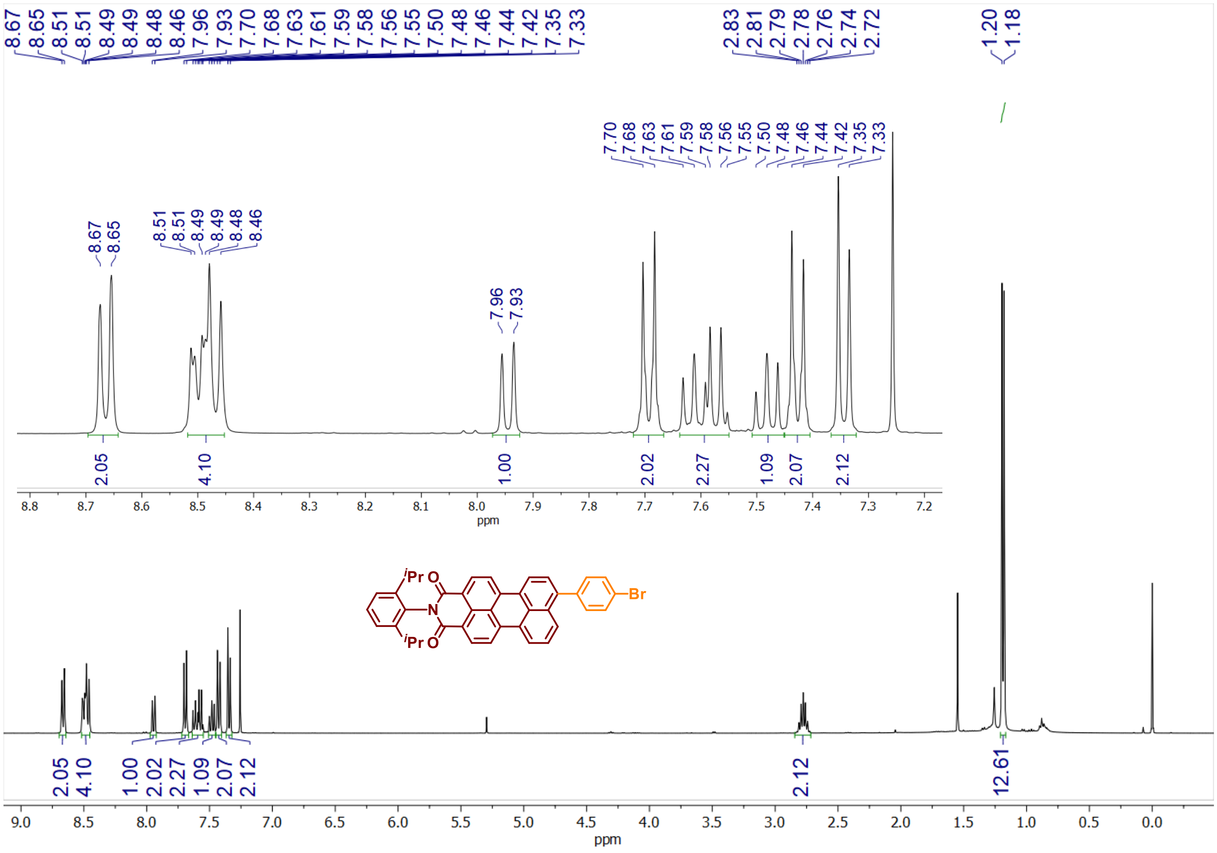

Figure S22. ^{1}H NMR spectrum of compound **P1Br** in $CDCl_3$.

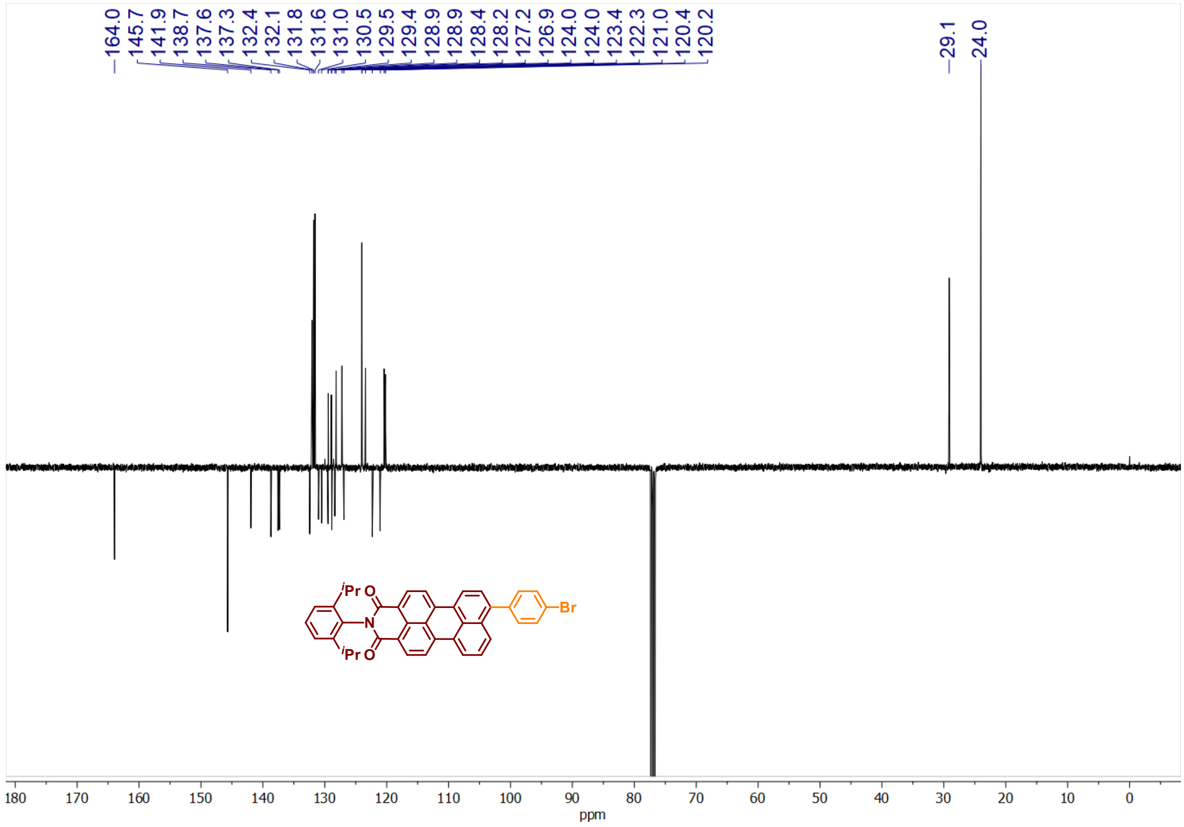

Figure S23. ^{13}C-APT NMR spectrum of **P1Br** in $CDCl_3$.

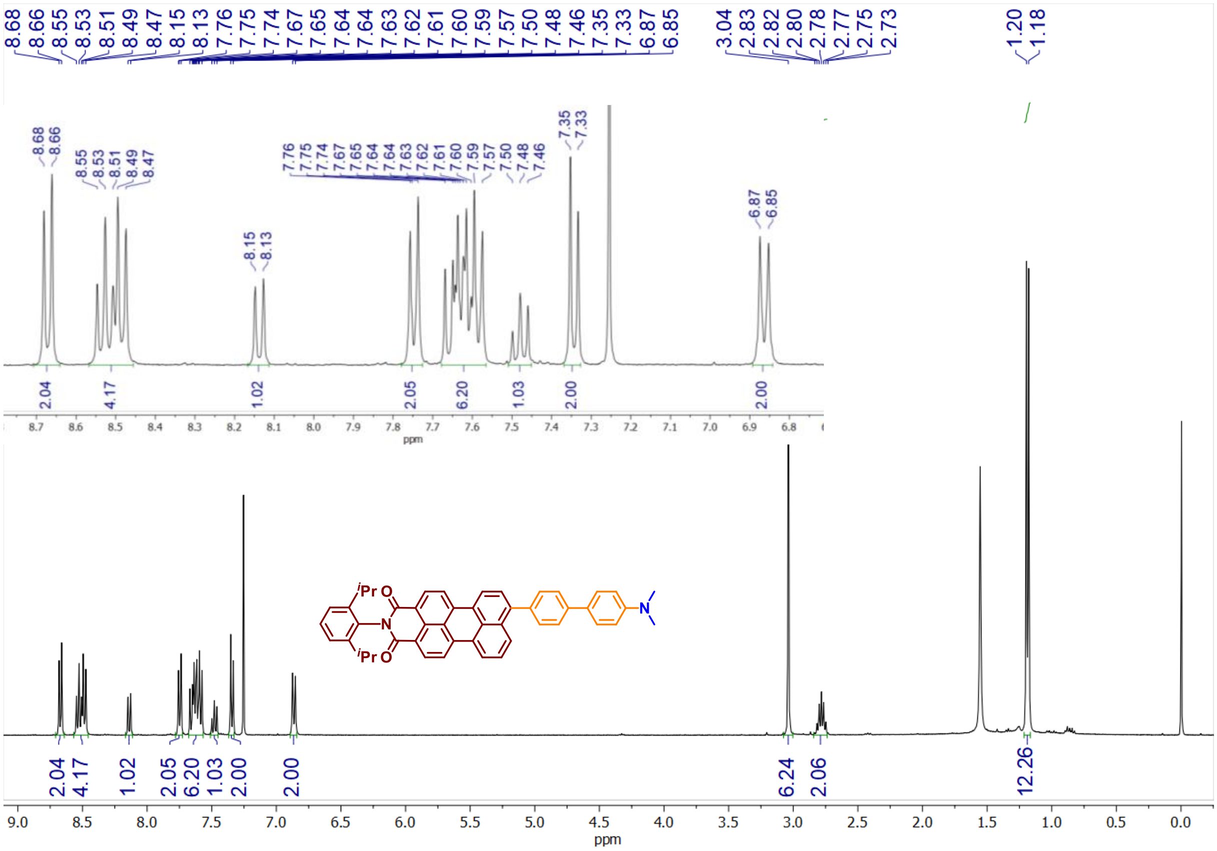

Figure S24. ^{1}H NMR spectrum of compound **P1** in $CDCl_3$.

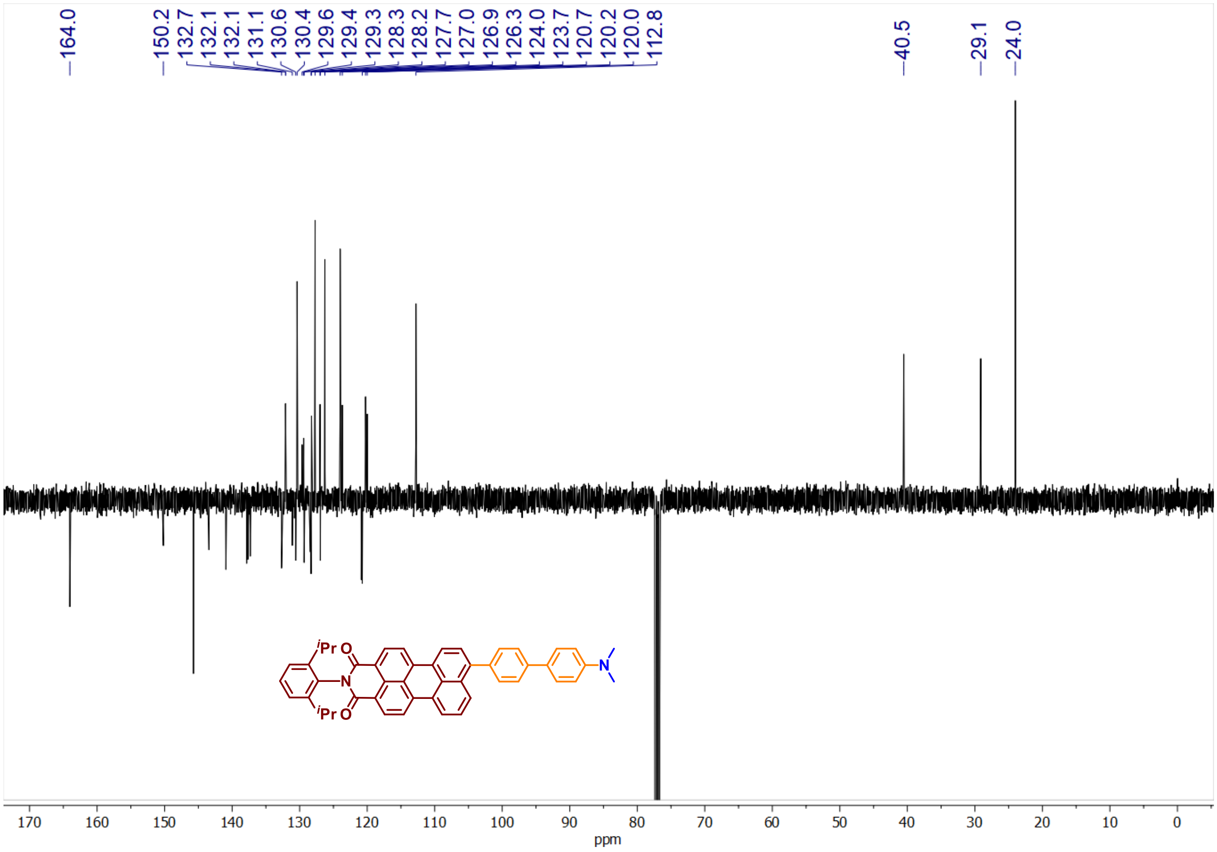

Figure S25. ^{13}C-APT NMR spectrum of **P1** in $CDCl_3$.

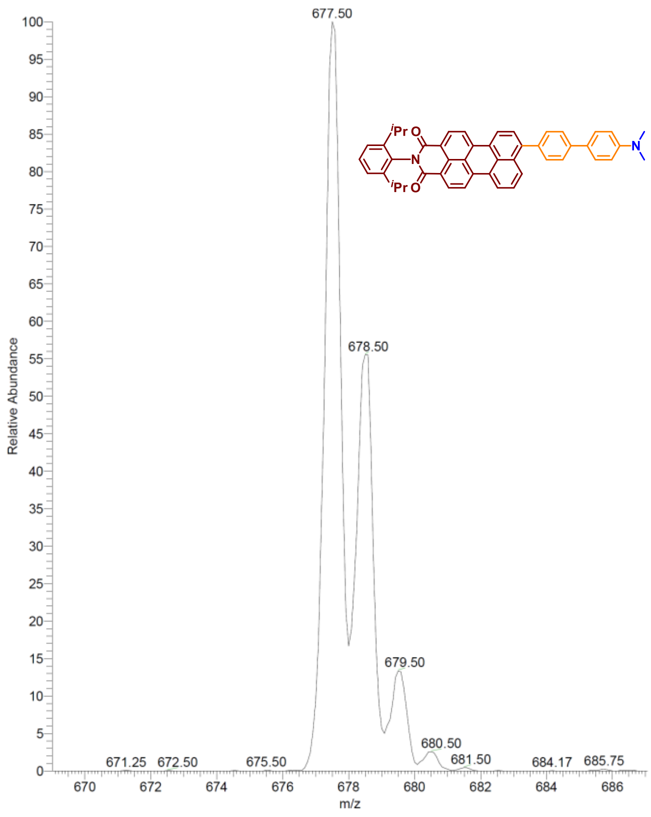

Figure S26. Mass spectrum of **P1**.

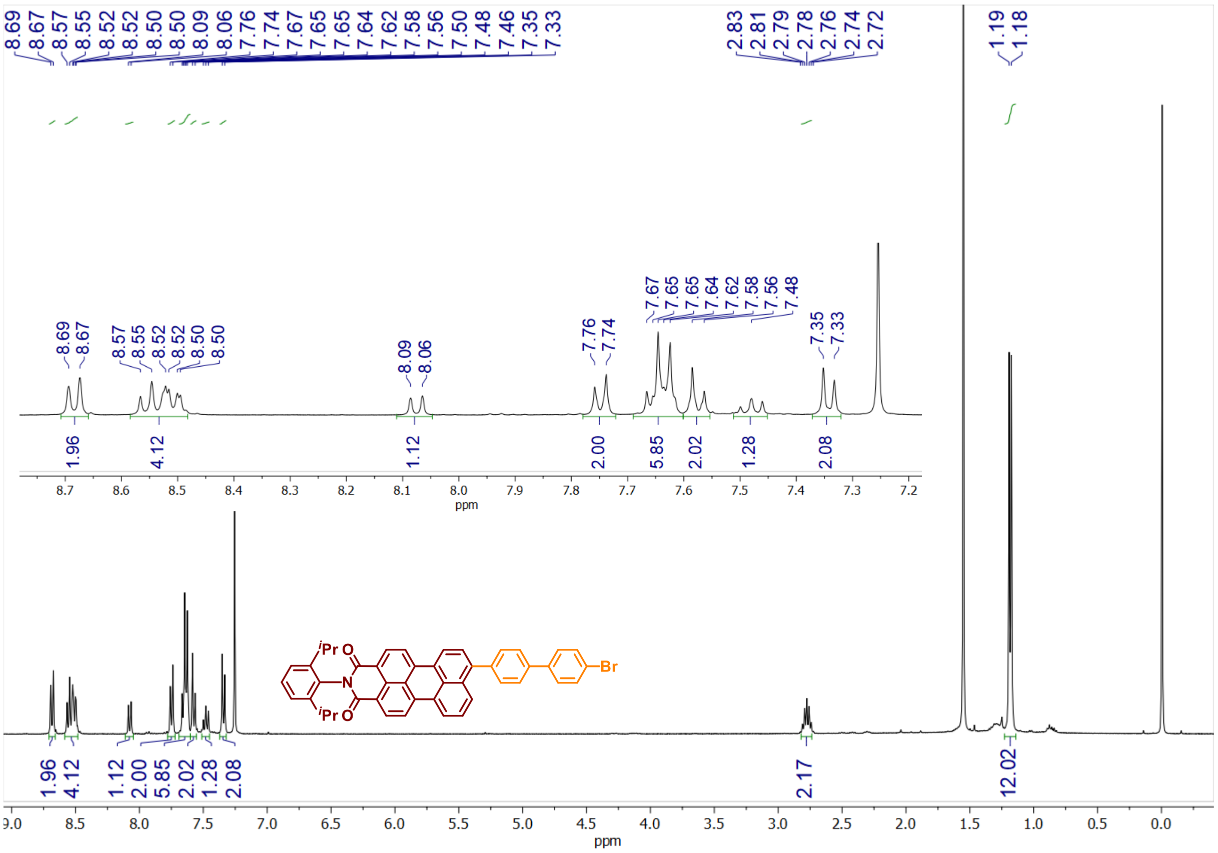

Figure S27. ^{1}H NMR spectrum of compound **P2Br** in $CDCl_3$.

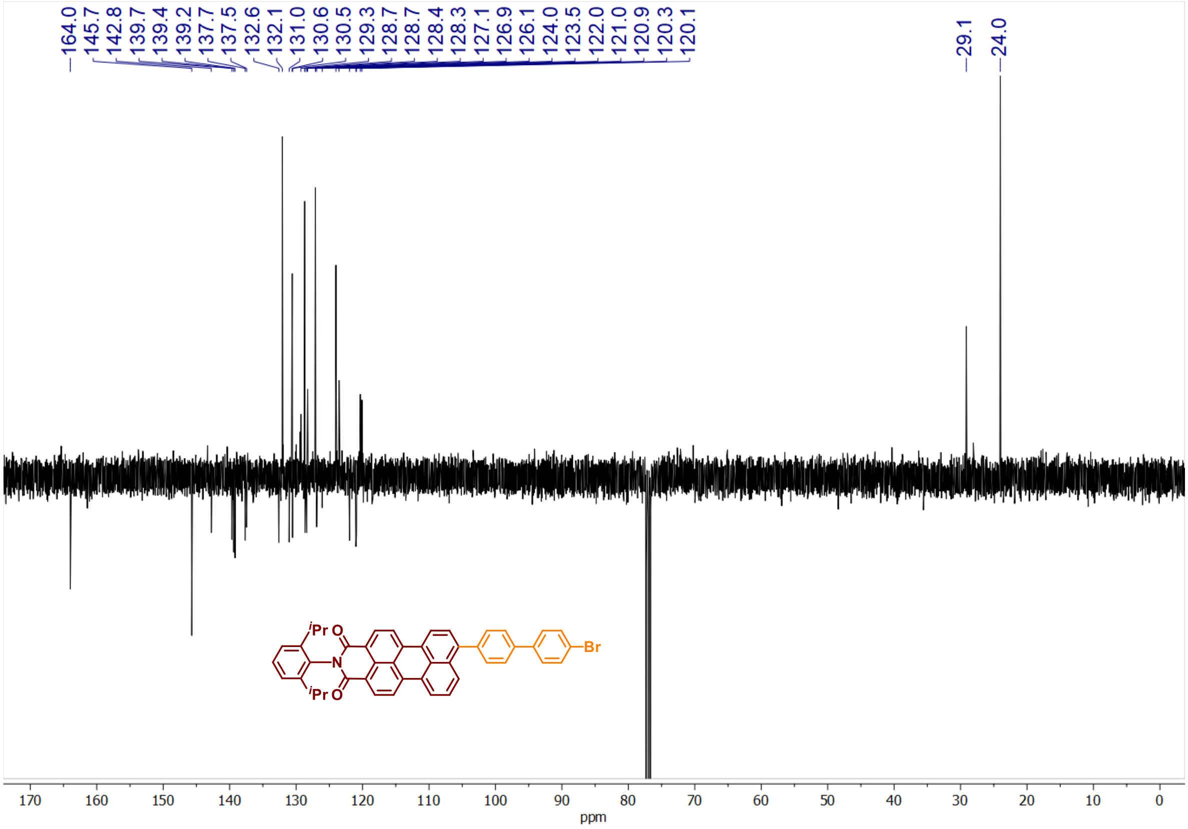

Figure S28. ^{13}C-APT NMR spectrum of **P2Br** in $CDCl_3$.

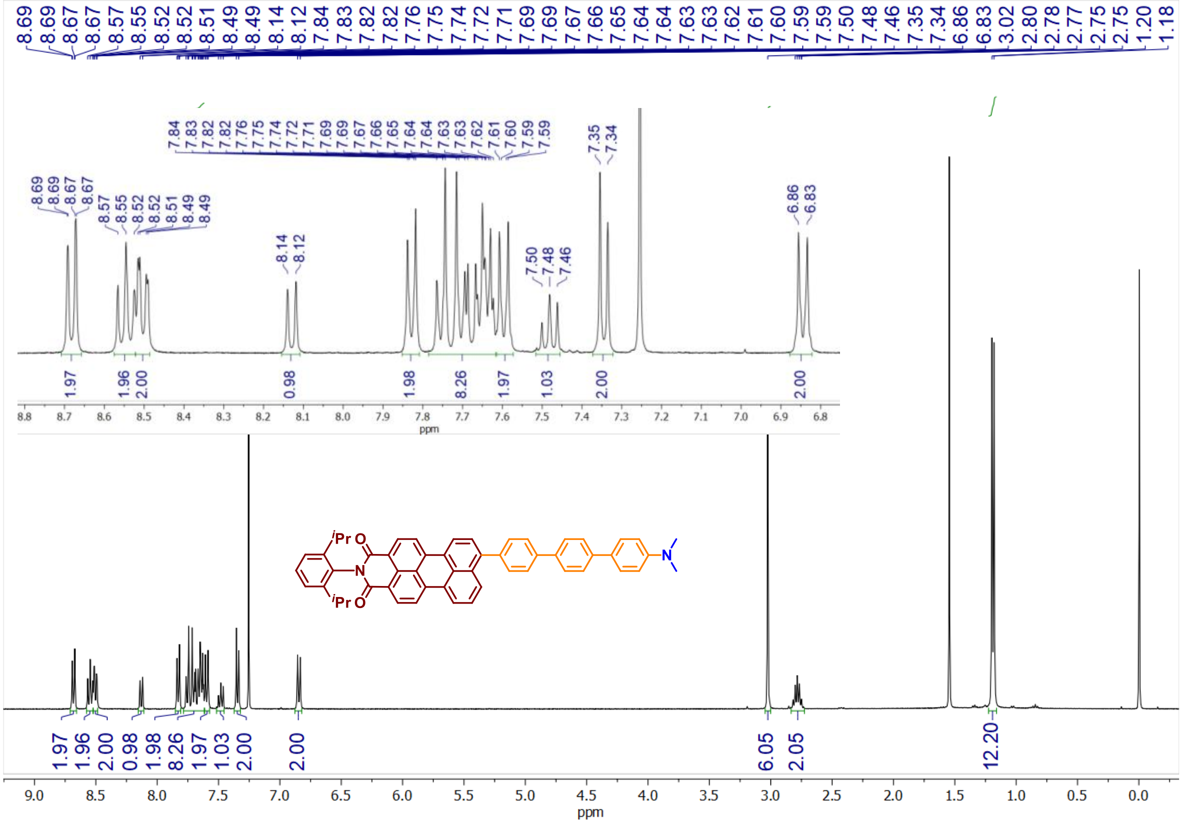

Figure S29. ^{1}H NMR spectrum of **P2** in $CDCl_3$.

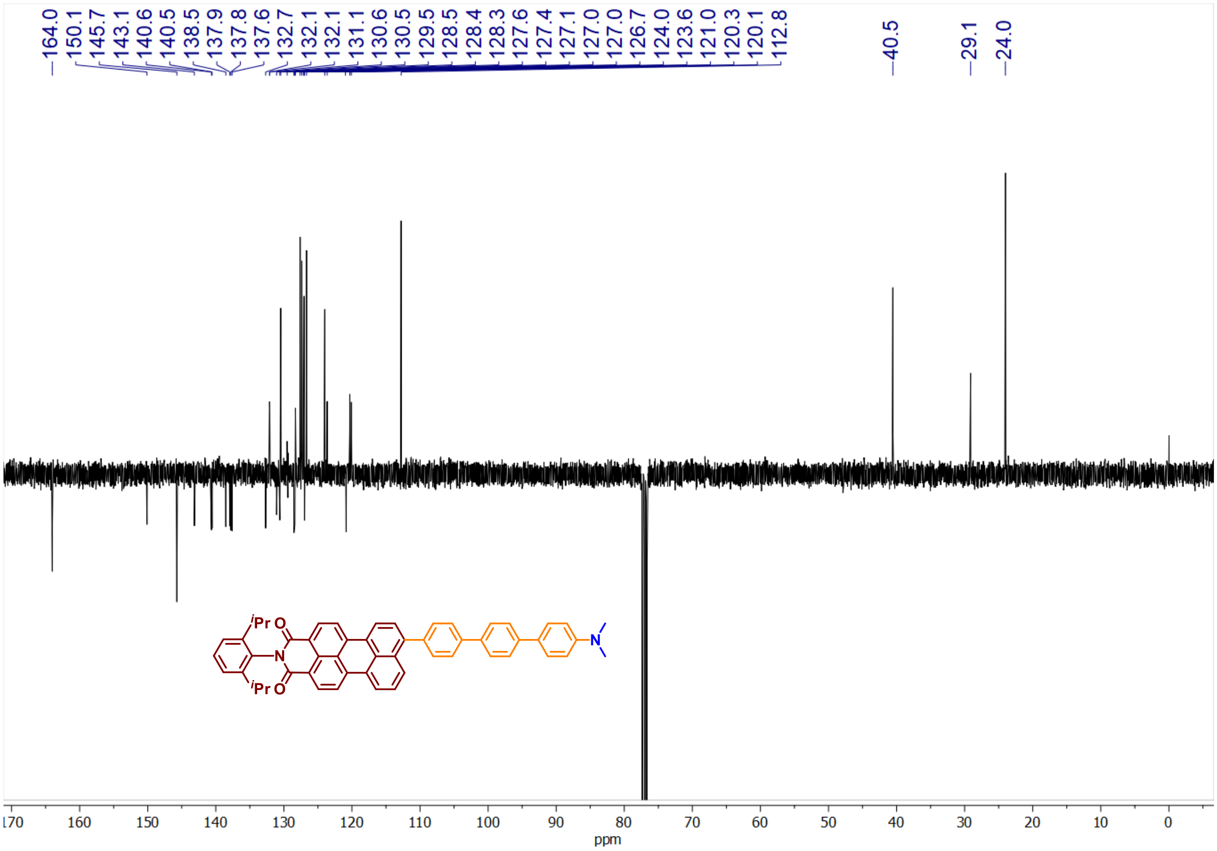

Figure S30. ^{13}C-APT NMR spectrum of **P2** in $CDCl_3$.

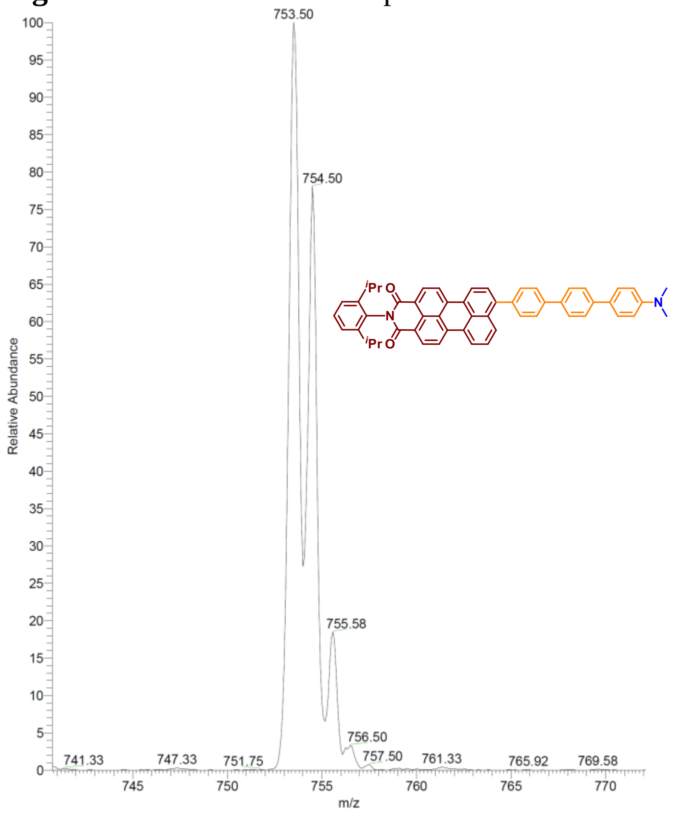

Figure S31. Mass spectrum of **P2**.

12. References

1. L. Feiler, H. Langhals and K. Polborn, *Liebigs Ann.*, 1995, **1995**, 1229-1244..
2. H. Wei, N. Jiang, N. Zhao, Y. Zhang and B. Gao, *Chin. J. Chem.*, 2014, **32**, 356-360.
3. T. Weil, M. A. Abdalla, C. Jatzke, J. Hengstler and K. Müllen, *Biomacromolecules*, 2005, **6**, 68-79.
4. T. Weil, E. Reuther, C. Beer and K. Müllen, *Chem. Eur. J.*, 2004, **10**, 1398-1414.
5. G. A. Crosby and J. N. Demas, *J. Phys. Chem.*, 1971, **75**, 991-1024.
6. P. G. Seybold, M. Gouterman and J. Callis, *Photochem. Photobiol.*, 1969, **9**, 229-242.
7. M. Taniguchi and J. S. Lindsey, *Photochem. Photobiol.*, 2018, **94**, 290-327.
8. R. Berera, R. van Grondelle and J. T. M. Kennis, Photosynth. Res., 2009, **101**, 105-118.
9. J. J. Snellenburg, S. P. Laptenok, R. Seger, K. M. Mullen and I. H. M. van Stokkum, J. Stat. Softw., 2012, **49**, 1-22.
10. A. Klamt and G. Schüürmann, J. Chem. Soc., Perkin Trans. 2, 1993, 799-805.
11. A. Kawaski, *Z. Naturforsch*., 2002, **57a**, 255-262.
12. E. Lippert, *Ber. Bunsenges. Phys. Chem.*, 1957, **61**, 962-975.
13. E. Sucre-Rosales, R. Fernández-Terán, N. Urdaneta, F. E. Hernández and L. Echevarria, *Chem. Phys.*, 2020, **537**, 110854.
14. A. Weller, *Z. Phys. Chem.*, 1982, **133**, 93-98.
15. Y. Liu, J. Zhao, A. Iagatti, L. Bussotti, P. Foggi, E. Castellucci, M. Di Donato and K.-L. Han, *J. Phys. Chem. C*, 2018, **122**, 2502-2511.
16. Y. Guo, Z. Ma, X. Niu, W. Zhang, M. Tao, Q. Guo, Z. Wang and A. Xia, *J. Am. Chem. Soc.*, 2019, **141**, 12789-12796.
17. R. A. Marcus, N. Sutin, Biochim. Biophys. Acta 1985, **811**, 265-322.
